# Effects of ivermectin treatment of backyard chickens on mosquito dynamics and West Nile virus transmission

**DOI:** 10.1371/journal.pntd.0010260

**Published:** 2022-03-25

**Authors:** Karen M. Holcomb, Chilinh Nguyen, Brian D. Foy, Michelle Ahn, Kurt Cramer, Emma T. Lonstrup, Asli Mete, Lisa A. Tell, Christopher M. Barker

**Affiliations:** 1 Davis Arbovirus Research and Training Laboratory, Department of Pathology, Microbiology, and Immunology, School of Veterinary Medicine, University of California, Davis, California, United States of America; 2 Center for Vector-borne Infectious Diseases, Department of Microbiology, Immunology and Pathology, Colorado State University, Fort Collins, Colorado, United States of America; 3 California Animal Health and Food Safety Lab, Department of Pathology, Microbiology & Immunology, University of California, Davis, California, United States of America; 4 Department of Medicine and Epidemiology, School of Veterinary Medicine, University of California, Davis, California, United States of America; University of Wisconsin Madison, UNITED STATES

## Abstract

**Background:**

Vector control strategies typically rely on pesticides to target mosquitoes involved in enzootic and zoonotic transmission of West Nile virus (WNV). Nevertheless, increasing insecticide resistance and a desire to reduce pesticide usage provide the impetus for developing alternative strategies. Ivermectin (IVM), an antiparasitic drug which is widely used in human and veterinary medicine, is a potential alternative for targeted control because *Culex* mosquitoes experience increased mortality following ingestion of IVM in bloodmeals.

**Methodology/Principal findings:**

We conducted a randomized field trial to investigate the impact of treating backyard chicken flocks with IVM in urban neighborhoods across Davis, California on mosquito populations and WNV transmission dynamics. We observed a significant reduction in WNV seroconversions in treated vs. untreated chickens, suggesting a reduction in WNV transmission intensity around treated flocks. We also detected a reduction in parity rates of *Cx*. *tarsalis* near treated vs. untreated flocks and increased mortality in wild mosquitoes following a bloodmeal on treated chickens (IVM serum concentration > 5ng/mL) vs. chickens with IVM serum concentrations < 5 ng/mL. However, we did not find a significant difference in abundance or infection prevalence in mosquitoes between treatment groups associated with the reductions in seroconversions. Mosquito immigration from surrounding larval habitat, relatively low WNV activity in the study area, and variable IVM serum concentrations likely contributed to uncertainty about the impact.

**Conclusions/Significance:**

Taken together, our results point to a reduction in WNV transmission due to the impact of IVM on *Culex* mosquito populations and support the ongoing investigation of oral administration of IVM to wild birds for local control of WNV transmission, although further work is needed to optimize dosing and understand effects on entomological endpoints.

## Author summary

Current mosquito control strategies aimed to prevent pathogen transmission to humans have limited ability to target mosquitoes involved in amplification and spillover transmission of pathogens like West Nile virus (WNV). Additionally, growing prevalence of insecticide resistance in mosquito populations limit the efficacy of these insecticide-based control strategies. Ivermectin (IVM) provides an alternative avenue for control by increasing the mortality of mosquitoes that ingest this drug in bloodmeals. Therefore, IVM treatment of avian species that account for the majority of mosquito bloodmeals during the WNV transmission season could be an effective control strategy. Building on pilot studies indicating the efficacy and feasibility of IVM-deployment for WNV control, we performed a randomized field trial to investigate the impact of IVM-treatment of backyard chickens on local population dynamics of *Culex* mosquitoes and WNV transmission. We were able to link changes in mosquito populations to reduction in WNV transmission, as measured by chicken seroconversions, through IVM-induced mortality in mosquitoes. However, further work is needed to identify the impact of treatment on mosquito abundance and infection prevalence to fully attribute observed changes to IVM administration. Overall, our results support IVM treatment as a potentially effective alternative to insecticide-based vector control strategies and one that can be used to target WNV transmission on the local scale.

## Introduction

West Nile virus (WNV) is a zoonotic mosquito-borne pathogen that can cause a potentially fatal, neuroinvasive disease in humans [1]. It is maintained in an enzootic cycle between birds [2,3] and bird-biting mosquitoes (predominantly in the genus *Culex*) [4], but can spill over to infect horses and humans, both of which are dead-end hosts susceptible to disease following infection [5]. WNV is the most widespread flavivirus with evidence of transmission on all continents except Antarctica [6] and the leading cause of mosquito-borne disease in the US [7]. While 80% of human infections are asymptomatic, approximately 20% result in a febrile illness and 1% in a neuroinvasive disease with manifestations including encephalitis, meningitis, and acute flaccid paralysis [8]. The severe form of the disease has an approximately 10% case fatality rate and often results in long-term physical and mental sequelae [9]. From 1999–2018, >50,000 cases and >2,300 associated deaths were reported in the United States [10], and the total number of infections is estimated to have exceeded 7 million [11]. The highest disease incidence occurs along the Great Plains, with a similar rate reported in some areas of California [12], where irrigated agriculture provides ample habitat for *Cx*. *tarsalis*, the primary WNV vector in the western United States [13], in proximity to avian hosts and humans [14,15].

Current WNV prevention strategies face several limitations. Because no licensed WNV vaccine exists for humans, prevention relies on mosquito control and personal protective measures (i.e., wearing long sleeves, avoiding dusk and dawn periods when WNV vectors are active, and using insect repellent) [16,17]. Control strategies primarily utilize chemical or microbial insecticides to manage mosquito populations in the larval or adult stages [18]. Larval control measures are generally preferred as a proactive strategy to target developing mosquitoes before they emerge from aquatic habitats [16]. Previous studies support the effectiveness of larviciding catch basins, a common larval *Culex* habitat, to reduce the abundance of larvae [19,20], but environmental conditions and suboptimal catch basin design can significantly reduce the efficacy [21,22], and larviciding alone is often insufficient to control mosquito populations and curb WNV transmission [23]. Ground-based adulticide applications can reduce target mosquito populations under ideal conditions, but estimates of the effects on WNV transmission are less consistent [24–27]. During periods of high epidemic risk, aerial applications of insecticides have been shown to rapidly reduce the abundance of WNV vectors [28], the abundance of infected mosquitoes [29–33], and human WNV cases in a treated area versus an untreated area [33], although such measures can be costly [34]. Also, efficacy varies widely due to differences in environmental conditions [35,36]. Overall, adulticide applications have limited precision to target bird-biting mosquitoes involved in enzootic and zoonotic transmission without disseminating pesticides over large areas, thereby increasing the possibility of non-target effects despite careful timing of applications to peak activity of target mosquitoes and lower activity of diurnal insects [18,37–40]. The efficacy of insecticide applications is also complicated by increasing levels of insecticide resistance in mosquito populations, which can render control measures less effective [41–43], prompting the development of alternative products and strategies.

Ivermectin (IVM), a widely used antiparasitic drug in human and veterinary medicine [44,45], provides the potential for targeted control by increasing the mortality of bird-feeding mosquitoes involved in maintenance and amplification of WNV. Mosquitoes that ingest IVM experience increased mortality [46,47], and few will likely survive long enough to take another bloodmeal at which pathogen transmission could occur, thus preventing future mosquito bites and blocking transmission. The mosquitocidal properties of IVM were first characterized in *Anopheles* mosquitoes in conjunction with mass drug administration campaigns that resulted in a reduction in malarial incidence [48,49].

Recently, the mosquitocidal applications of IVM have been investigated in *Culex* mosquitoes for controlling WNV transmission. It was hypothesized that targeting the common avian species that account for the majority of *Cx*. *tarsalis* bloodmeals during the WNV transmission season could act as an effective WNV control strategy [46]. Using pilot laboratory and field-based trials, Nguyen et al. [46] demonstrated the feasibility and effectiveness of developing IVM-treated birdfeed as a novel WNV transmission control strategy. IVM use in birds is primarily extra-label (i.e., use of an approved drug in a manner not in accordance with the approved labeling, but meets the conditions set forth by the Animal Medicinal Drug Use Clarification Act of 1994 [50] and the U.S. Food and Drug Administration). IVM has been used widely and effectively to treat a variety of avian parasites in taxa including falcons, budgerigars, and chickens [51–54]. Nguyen et al. [46] observed no toxicity in chickens and doves fed exclusively on IVM-treated feed (200 mg IVM/kg feed) for 3–10 days and demonstrated the mosquitocidal activity of the blood of these orally treated birds [46].

While not involved in the enzootic transmission cycle of WNV, chickens are a common bloodmeal source for WNV vectors, being preferentially bitten over other species within 50 m of flocks [55,56]. As chickens are refractory to disease, they are often used as WNV sentinels [57]. Also, in contrast to wild birds, backyard chickens remain in a single location throughout the WNV season, providing a consistent location from which to expose biting mosquitoes to IVM. Thus, backyard chickens are an ideal study species for initial deployment of IVM. Our study, conducted in suburban neighborhoods across Davis, California, aimed to determine whether IVM delivered through backyard chicken flocks can suppress the abundance of WNV-infected mosquitoes and transmission of WNV as measured by chicken seroconversions. To this end, we monitored entomological indices of *Cx*. *tarsalis* populations (i.e., abundance, infection prevalence, and parity) as well as both serum IVM concentrations and WNV seroconversions in IVM-treated and untreated chickens. We also assessed IVM-induced mortality of wild-caught *Cx*. *tarsalis* following a bloodmeal on treated chickens to connect differences in mosquito population and infection transmission dynamics to IVM treatment. This study expanded upon previous pilot studies and paralleled a concurrent field trial in northern Colorado.

## Methods

### Ethics statement

This study was carried out in strict accordance with the UC Davis Institutional Animal Care and Use Committee (IACUC) Protocol #20980 that was reviewed and approved on February 6, 2019. The UC Davis IACUC adheres to the Office of Laboratory Animal Welfare Health Research Extension Act of 1985 (Public Law 99–158) as well as the United State Department of Agriculture’s Animal Welfare Act. UC Davis is accredited by the Association for Assessment and Accreditation of Laboratory Animal Care, International (AAALAC) and has an Animal Welfare Assurance (number A3433-01) on file with the Office of Laboratory Animal Welfare (OLAW).

### Colony mosquito membrane feeding assays

To confirm previous findings of the susceptibility of *Cx*. *tarsalis* to IVM, we performed artificial membrane feedings over a range of oral IVM doses using the Kern National Wildlife Refuge (KNWR) colony established in 2002 from *Cx*. *tarsalis* collected at the Kern National Wildlife Refuge (35.7360° N, 119.5979° W), in Kern County, California. *Cx*. *tarsalis* were reared under consistent insectary conditions (temperature 24°C, relative humidity 40–60%, photoperiod 14L:10D). Larvae were reared in plastic trays with approximately 300–400 larvae in approximately 750 mL of water and fed ground Tetramin fish food (Spectrum Brands Pet, Blacksburg, VA, USA) daily until pupation. Adults were housed at approximately 300 per cage (61 x 61 x 61 cm) with constant access to 10% sucrose solution until allocation into 3.97 L (1 gal) plastic cartons with screen tops for bioassays. For mosquito bioassays, we added IVM (Sigma Aldrich 18898, PubChem Substance ID: 24278497) in heparinized sheep blood (Hemostat Laboratories, Dixon, CA, USA) at serial dilutions (600, 300, 150, 75, 37.5, and 0 ng IVM/mL) for artificial membrane feeding. Approximately 70 adults were allocated into each treatment group. Following blood feeding, fully-engorged females were collected with a hand aspirator and held for nine days in the same insectary conditions. Mosquito mortality was recorded every 24 hours. The lethal concentration resulting in 50% mortality (LC_50_) was calculated using probit regression analysis (R statistical software, version 4.0.2 [58]).

### Chicken flock field sites

**Fig 1:**
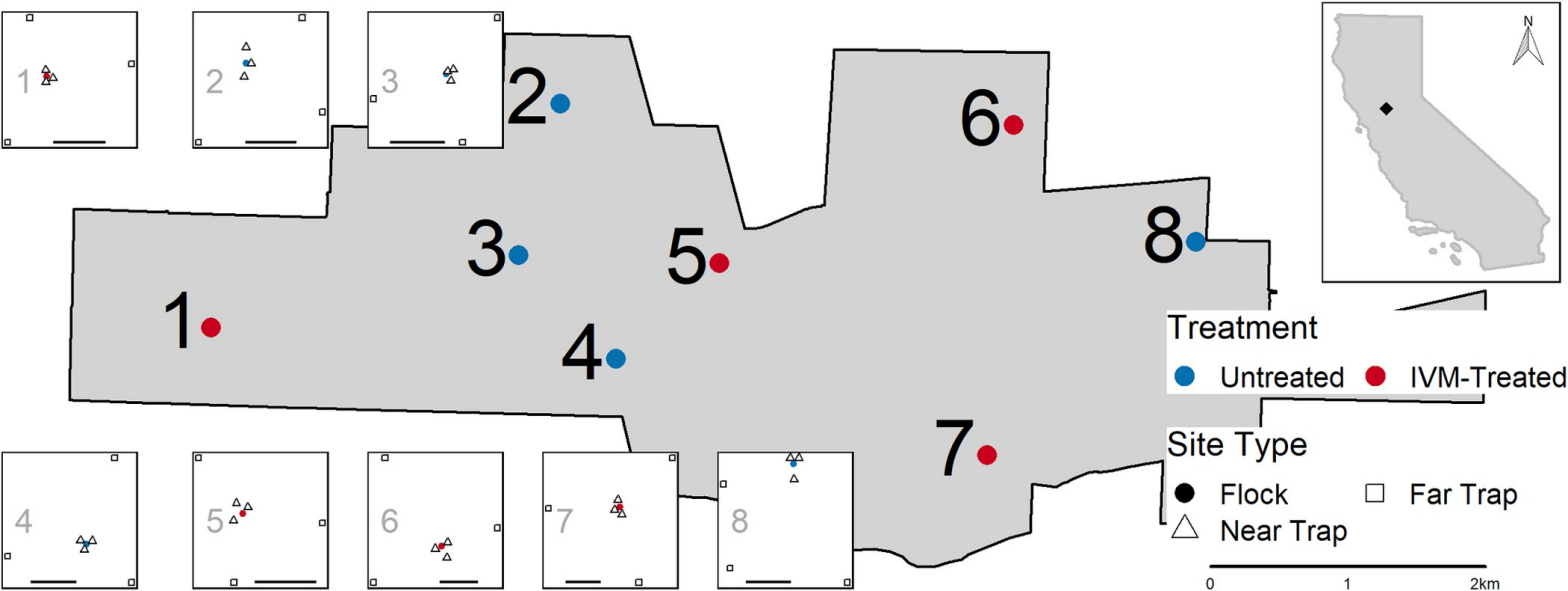
Location of ivermectin (IVM)-treated (red) and untreated (blue) chicken flocks and CO2-baited mosquito traps in Davis, California. Numbered insets illustrate the arrangement of dry-ice baited CO2-traps around each flock, with three traps within 10m (triangle) and three traps approximately 150m from the flock (square). Bar in each inset indicates 100m. Top right inset illustrates the location of the study site in relation to the state of California. Geographic boundaries for the state of California and the city of Davis were obtained from the 2020 TIGER/Line shapefiles for States and Places, respectively, provided by the United States Census Bureau (https://www.census.gov/cgi-bin/geo/shapefiles/index.php).

We placed eight flocks—four IVM-treated and four untreated controls—of six chickens per flock in coops in backyards across Davis, California from June 28—Sept 20, 2019 (Fig 1). Locations were chosen to achieve broad spatial coverage of the range of backyard environments in suburban areas of Davis (e.g., age of house, proximity to apartment buildings or natural spaces, variety of predominant vegetation) and in backyards of homeowners willing to host chickens for the duration of the study. We randomly assigned treatment status to flocks. We also placed 24 chickens in three coops at the UC Davis south campus facilities >2 km south of Davis city limits as an untreated reserve flock that could serve as a source of replacement chickens for other backyard flocks if needed. We used 16-month-old female Lohmann Brown chickens, which were housed in the Innovation Pet Chicken Homestead Coop (Tractor Supply, Brentwood, TN, USA), replacing the original 1.27 cm mesh sides with 2.54 x 2.54 cm welded wire mesh (YardGard, North Plains, OR, USA) to permit mosquitoes to access the chickens freely for blood-feeding and exclude predators. We obtained chickens from a pasture poultry training and outreach program at the University of California, Davis, which teaches homeowners about proper care and management of pasture and free-range poultry flocks.

Our study design (number of flocks per treatment group and number of mosquito traps per week per flock) was based on assumptions of weekly average abundance and infection prevalence based on historical mosquito collections from Davis [59]. The calculations followed those of [60] for a cluster-randomized trial. Assuming an average of 92.5 *Cx*. *tarsalis* per week for 13 weeks and six traps per flock location, four flocks per treatment group would result in 80% power (at an alpha of 0.05) to detect a 50% reduction in infection prevalence (i.e., 7/1,000 in untreated vs. 3.5/1,000 in treated groups). We also assumed a normal distribution of infection prevalence in untreated flocks with a mean of 7/1,000 and a standard deviation of 1.5/1,000, leading to a coefficient of variation of 1.5/7 between flocks in each treatment group.

### Chicken care and monitoring

Treated flocks received IVM daily via free choice ingestion of medicated feed (200 mg IVM/kg feed) from July 8—Sept 18, 2019. We mechanically mixed powdered IVM into chicken feed daily (1:40 ratio of DuMOR 16% Poultry Layer Crumbles and DuMOR grit, Tractor Supply, Brentwood, TN, USA). All flocks received a total of 0.907 kg of feed (mean of 0.151 kg per chicken) daily.

We weighed chickens twice during the study to assess any differences in flocks between treatment groups. We used a spring scale (analog linear 3kg hanging scale, Chatillon & Sons, Largo, FL, USA) to obtain weights. During the weighing process, chickens were placed in a plastic bucket (1 gal) and the weight of the bucket was removed from the scale reading to obtain the final weight. Difference in weight and change in weight between timepoints across treatment groups was assessed using t-tests.

We monitored for WNV seroconversions in all chickens and IVM serum concentrations in samples from treated chickens every 1–2 weeks throughout the study, taking all blood samples at similar times in the morning. We took samples from untreated chickens 2–4 times during the study to confirm no accidental introduction of IVM to untreated control flocks. We obtained blood samples from a comb prick and/or the brachial vein. We initially used a comb prick to obtain a small blood sample for WNV surveillance, but a brachial bleed reduced the handling time so all blood samples after week four were acquired via brachial bleeds.

For WNV serology, we soaked a 1.27 cm wide filter paper strip with blood, either from the comb following piercing with a standard lancet [17] or from the blood obtained from the brachial bleed. Blood samples on filter paper were submitted to the California Department of Public Health for testing for IgG antibodies to flaviviruses (West Nile, western equine encephalomyelitis, and St. Louis encephalitis viruses) using an enzyme immunoassay [61,62]. A western blot was used to confirm that flavivirus-positive sera were attributable to WNV and not St. Louis encephalitis virus. WNV seroconversions in IVM-treated vs. untreated flocks were analyzed using Kaplan-Meier survival curves and compared using the Mantel-Haenszel test in R [58] (version 4.0.2; survival package version 3.1–12 [63]).

For quantification of IVM, we collected whole blood samples into serum tubes (Greiner Bio-One Serum Clot Activator Tubes, Fisher Scientific, Pittsburgh, PA, USA), gently inverted three to five times, and held at ambient temperature to coagulate. Following coagulation, we centrifuged the blood at 1,800 RPM for 10 minutes at 4°C and removed the serum. Serum samples were stored approximately 18 months at -80°C until testing to quantify IVM using high-performance liquid chromatography (HPLC)-fluorescence. IVM was first extracted from serum and derivatized after methanol precipitation, following [46,64,65] with the modification that 50 μL of serum was added to 400 μL of methanol prior to vortexing. A Waters 700 autosampler system was then used for quantification as previously described [46]. Briefly, a mobile phase of acetonitrile/water (3:1, v/v) was pumped through a C8 column (Waters, XBridge BEH C8 XP, 130 Å, 2.5 μm, 3.0x100 mm) at a rate of 0.45 mL/min and 40 μL of derivatized sample was injected by the autosampler. Excitation and emission spectra were 365 and 470 nm, respectively.

### Mosquito monitoring and indices

#### Entomological indicators of WNV risk

We collected mosquitoes weekly to estimate *Culex* abundance, infection prevalence, and parity near (≤10 m) and far (~150 m) from each coop location using an array of six CO_2_-baited traps (CDC miniature light trap (Model 512) with light bulb removed, John W. Hock CO, Gainesville, FL, USA) per location, three near and three far (Fig 1). We chose the near and far distances of trap placements based on previous findings that chickens were bitten preferentially within 50 m of flocks, but not at greater distances [55,56]. Traps were placed in yards, greenbelts, and parks, aiming to maximize similarity of environmental contexts of trap sites across coop locations. We placed traps between the hours of 14:30–18:00 PM and picked them up following morning between 07:30–10:00 AM. Each week, half were run Mon-Tue and the other half Wed-Thu. Collected mosquitoes were immobilized with triethylamine [66] and identified by species and sex [67]. *Cx*. *tarsalis* and *Cx*. *pipiens* females were pooled separately for WNV testing (pools up to ~50 each). Mosquito pools were stored on dry ice and submitted to the Davis Arbovirus Research and Training (DART) lab for testing by multiplex RT-qPCR. Each pool was screened for West Nile virus, western equine encephalomyelitis virus, and St. Louis encephalitis virus [68,69]. Positive pools with Ct scores <35 were confirmed by singleplex RT-qPCR with a different set of virus species-specific primers and probes. If >50 *Cx*. *tarsalis* females were collected from a trapping site, we randomly removed 20% (up to 30 individuals) for parity dissection. We calculated vector index [70], which is the product of relative abundance and infection prevalence, to estimate the number of infected mosquitoes present at each distance for treated and untreated locations. We used ANOVA to compare total abundance, infection prevalence, and vector index separately between near and far trap locations for treated and untreated locations across the study period (car package version 3.0–8 [71] in R software [58], version 4.0.2). In addition to the IVM treatment and distance variables of interest, all ANOVA models included terms for week to account for changes over time.

#### Mosquito age structure

**Fig 2:**
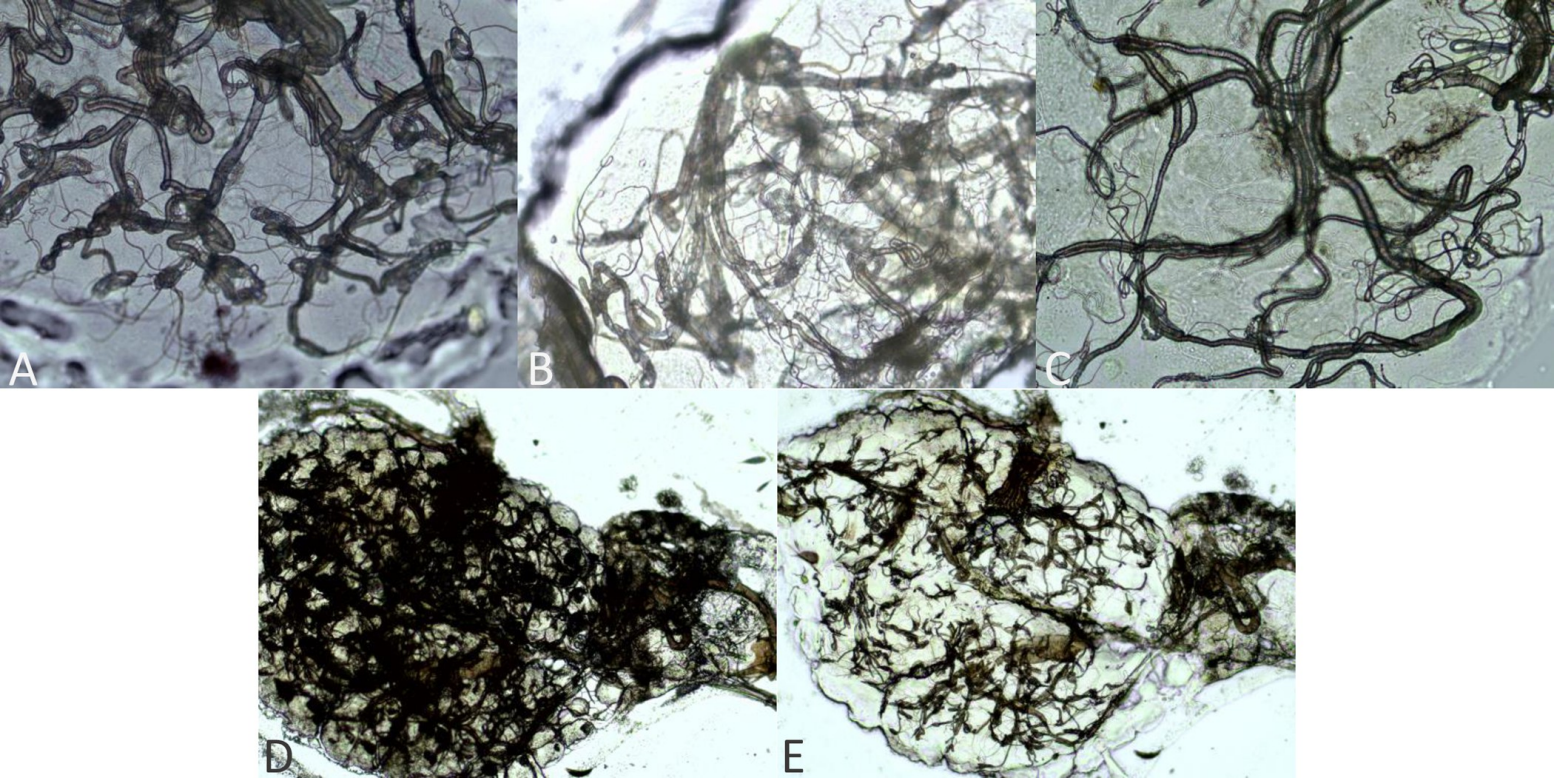
Categories used for ovarian grading in *Cx*. *tarsalis*. Parity status graded as A) nulliparous with all tightly wound skeins, B) intermediate with a combination of tight and loose skeins, or C) parous with all unwound skeins. D) Dark mass observed in some ovaries, likely due to egg protein, which is E) removed with washing with deionized water. Ovarian mounts presented at 400x magnification (A-C) or 100x magnification (D-E).

Following previously described ovarian tracheation techniques [72,73], we dissected mosquito ovaries in a drop of deionized (DI) water on a glass slide under a stereomicroscope and mounted them on slides, allowing them to dry before storing them in slide boxes. If we were unable to complete mosquito dissections on the day of trap collections, they were stored at 4°C for up to two days until dissections could be completed. After drying, slides were stored at room temperature until they could be examined. All slides were read using a compound microscope independently by two researchers (KMH and ETL) who were blinded to the treatment status. Any discrepancies in grading were resolved by mutual consent following reobservation. If a reticulated pattern or dark mass obscured the ovary (Fig 2D) [74], likely due to egg protein [75], we washed slides briefly with DI water and air-dried before re-examining (Fig 2E). We classified ovaries as nulliparous when all skeins were tightly coiled (Fig 2A), parous when all skeins were completely unwound (Fig 2C), and intermediate when a combination of tightly wound and unwound skeins were observed, or if all skeins appeared loose but not fully unwound (Fig 2B) [74–76]. We included this third category due to high rates of autogeny in *Cx*. *tarsalis* previously reported in California’s Central Valley [77,78]; autogenous females lay a smaller than average egg batch prior to the first bloodmeal, resulting in an intermediate appearance that cannot be accurately assigned prior to the first bloodmeal [75]. Following subsequent bloodmeals, ovaries of autogenous and anautogenous mosquitoes are indistinguishable. We first compared the overall parity rates (i.e., proportion of parous mosquitoes) across distances for each treatment group using χ^2^-tests (R software [58], version 4.0.2) and then examined the number of parous vs. nulliparous or intermediate mosquitoes between treatment groups and distances with mixed effects logistic regression, using week as a random intercept (lme4 package version 1.1–23 [79] in R [58]). Comparisons between all treatment-distance pairings were based on the Wald test for regression coefficients (aod package version 1.3.1 [80] in R [58]). For both the χ^2^-test and mixed-effects model, we combined the intermediate and nulliparous categories to obtain a conservative estimate of differences in age structure.

#### Mortality of wild-caught mosquitoes after IVM feeding

At the termination of the study (Sept 16), we fed wild-caught adult *Cx*. *tarsalis* females on one, randomly selected chicken from each flock to assess the mosquitocidal activity of the blood of treated chickens vs. untreated chickens for wild mosquitoes. *Cx*. *tarsalis* were collected using CO_2_-baited EVS traps on a single night within the nearby Yolo Bypass Wildlife Area (38.5266°N, 121.6461°W), a 65-km^2^ area consisting primarily of rice (*Oryza sativa* L.) fields and managed wetlands. We allowed approximately 700 wild-caught *Cx*. *tarsalis* an opportunity to feed on each chicken overnight. Each chicken was placed within a lidded plastic bin (73.03 x 40.64 x 46.36 cm) inside a secondary 120 x 60 x 60 cm mesh enclosure (BugDorm-6M60, Taiwan), set within the same backyard as the flock from which it was taken. The sides of the plastic bin were cut out, leaving 2.5–5 cm borders around all sides, and the open plastic bins were wrapped in 2.54 cm-square chicken wire that was secured to the bin’s frame with zip ties. Our design allowed mosquitoes introduced into the container to have free access to the chicken, but also have ample resting locations out of reach of the chicken within the BugDorm. In the morning, all mosquitoes were collected from the BugDorms using a hand aspirator and sorted in the lab; blood-fed mosquitoes from each flock location were placed together in an individual 3.79 L (1 gal) plastic carton with screen top, provided a sugar water-soaked cotton ball or wick, and held in standard insectary conditions for eleven days. We recorded mortality and removed dead mosquitoes daily. We analyzed the survival of wild mosquitoes fed on treated vs. untreated chickens using Kaplan-Meier survival curves and compared using the Mantel-Haenszel test. We also compared mosquito survival stratified relative to the final IVM serum concentration in chickens (either above or below the limit of quantification, LOQ) using Kaplan-Meier survival curves and compared using the Mantel-Haenszel test. Survival analyses were performed using the survival package version 3.1–12 [63] in R (version 4.0.2 [58]).

### Safety of IVM ingestion

To assess any effects of prolonged oral exposure to IVM in birds, we necropsied twelve chickens upon completion of the field study (Sept 13); three randomly selected per flock from two treated and two untreated flocks. The pathologist was blinded to the treatment status of the chickens. Samples were taken for histological examination (brain, peripheral nerves, skeletal muscle, heart, lungs, trachea, liver, kidney, ovary, pancreas, and intestines). During the necropsy, a gross examination was made to assess the overall health, tissue status, and presence of parasites. At this time, we also obtained whole blood for WNV serology and IVM quantification. Whole blood was submitted directly to California Department of Public Health for WNV serology. Fisher’s exact test was used to compare the distribution of pathological and histological findings at necropsy between treated and untreated groups (R software, version 4.0.2 [58]).

## Results

### Bioassay with colony *Cx*. *tarsalis*

In the initial laboratory trial, laboratory-reared *Cx*. *tarsalis* mosquitoes (KNWR colony) were found to be susceptible to IVM. We observed sharp reductions in survivorship following ingestion and 87–100% mortality within three days for doses >75 ng/mL (S1 Fig). The estimated LC_50_ for the first three days post-bloodmeal was 66.03 ng/mL.

### Chicken WNV seroconversions

Due to delays in chicken availability, we obtained baseline blood samples from all chickens on the same day the chickens were placed in backyards. After serological testing, untreated chickens that were initially seropositive at baseline were replaced with immunologically naïve chickens from our reserve flock (replaced on Jul 17), but seven treated seropositive chickens could not be rehoused following IVM treatment. Therefore, total numbers of chickens were equal (*n* = 6) in all study flocks, but a total of seven seropositive chickens remained in treated flocks, resulting in 3–5 seronegative chickens per treated flock at the start of the study.

**Fig 3:**
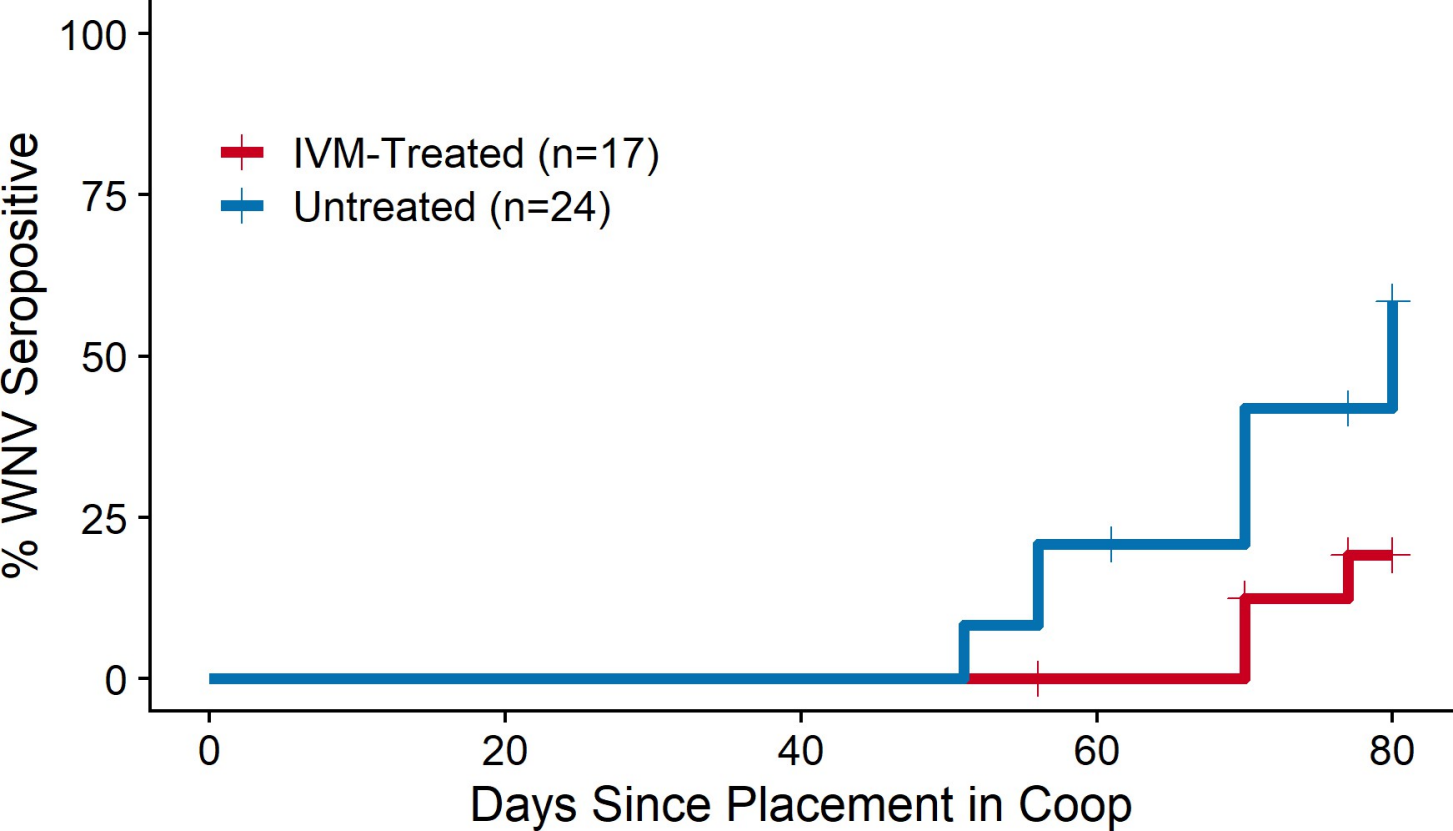
Reduced West Nile virus (WNV) seroconversions in ivermectin (IVM)-treated vs. untreated chicken flocks. Four flocks per treatment group. Chickens seropositive at baseline were excluded from the analysis.

At the end of the study, accounting for the timing of replacement of seropositive chickens (i.e., variation in duration of risk periods), fewer chickens seroconverted in treated flocks (3/17, 18%) than in untreated flocks (11/24, 46%), and these seroconversions occurred later in the season compared to untreated flocks, resulting in significantly lower WNV transmission to chickens at treated locations (Fig 3, χ^2^ = 4.7, *P* = 0.03).

### IVM serum concentrations in chickens

**Fig 4:**
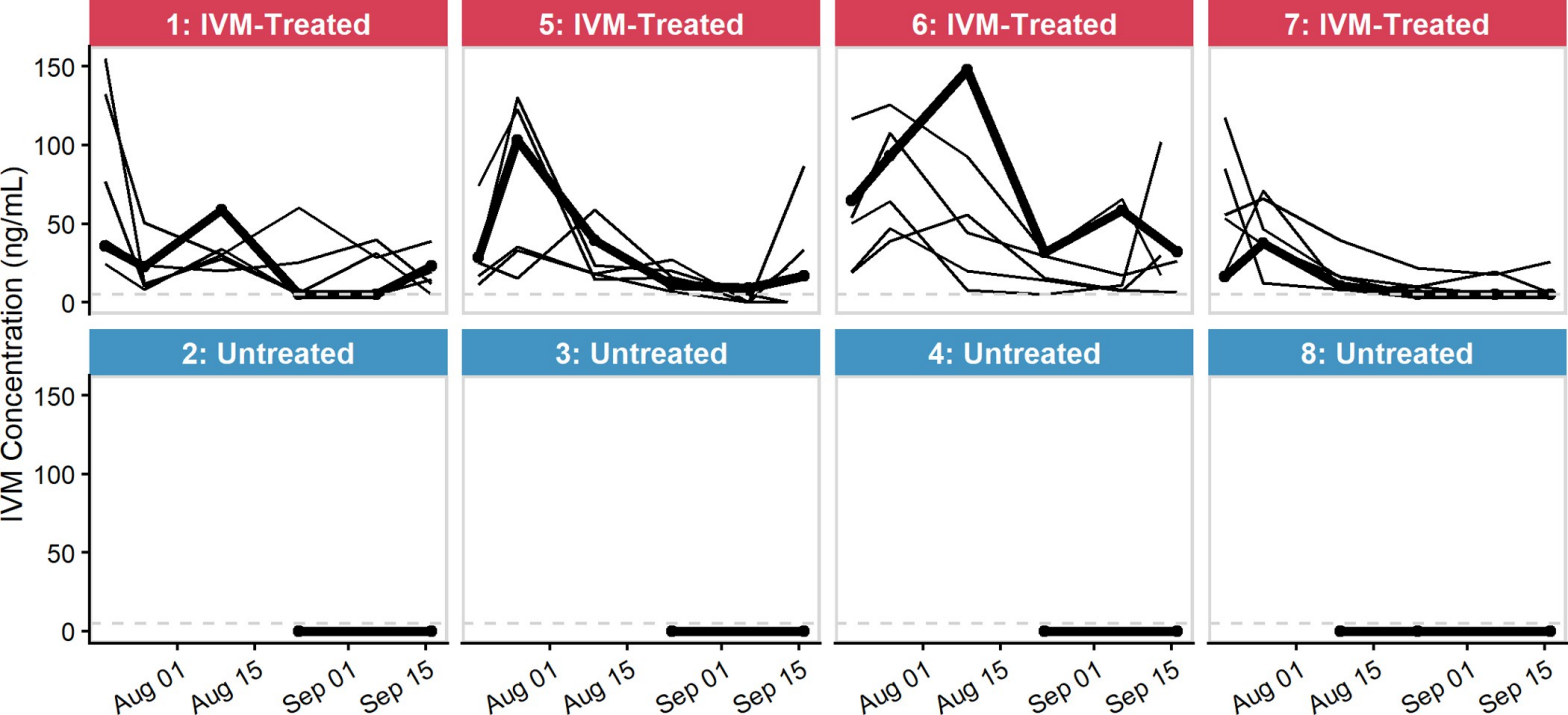
Ivermectin (IVM) serum concentrations (ng/mL) in treated and untreated chickens. Grey dashed line indicates the limit of quantification (LOQ, 5 ng/mL). Bold line indicates chicken used in mosquito bioassay at end of study.

In treated chickens, IVM serum concentrations ranged from <5–155.2 ng/mL, with an average concentration of 33.1 ng/mL (Fig 4). Ivermectin serum concentration generally peaked early in the study period (max 155.2 ng/mL; mean initial concentration 54.9 ng/mL) and decreased to lower concentrations during the remainder of the study period (mean final concentration 22.6 ng/mL). All samples with unquantifiable IVM concentrations in treated chickens (five samples from four chickens) were from a single flock (coop 7). All of these chickens had quantifiable levels of IVM at other times during the study. The assay’s limit of quantification (LOQ) was 5 ng/mL.

Serum samples from untreated chickens had no IVM detected throughout the study period, confirming that there was no accidental cross-contamination of IVM feed between flock locations.

### Mortality in field-collected *Cx*. *tarsalis* following blood-feeding on IVM-treated chickens

**Fig 5:**
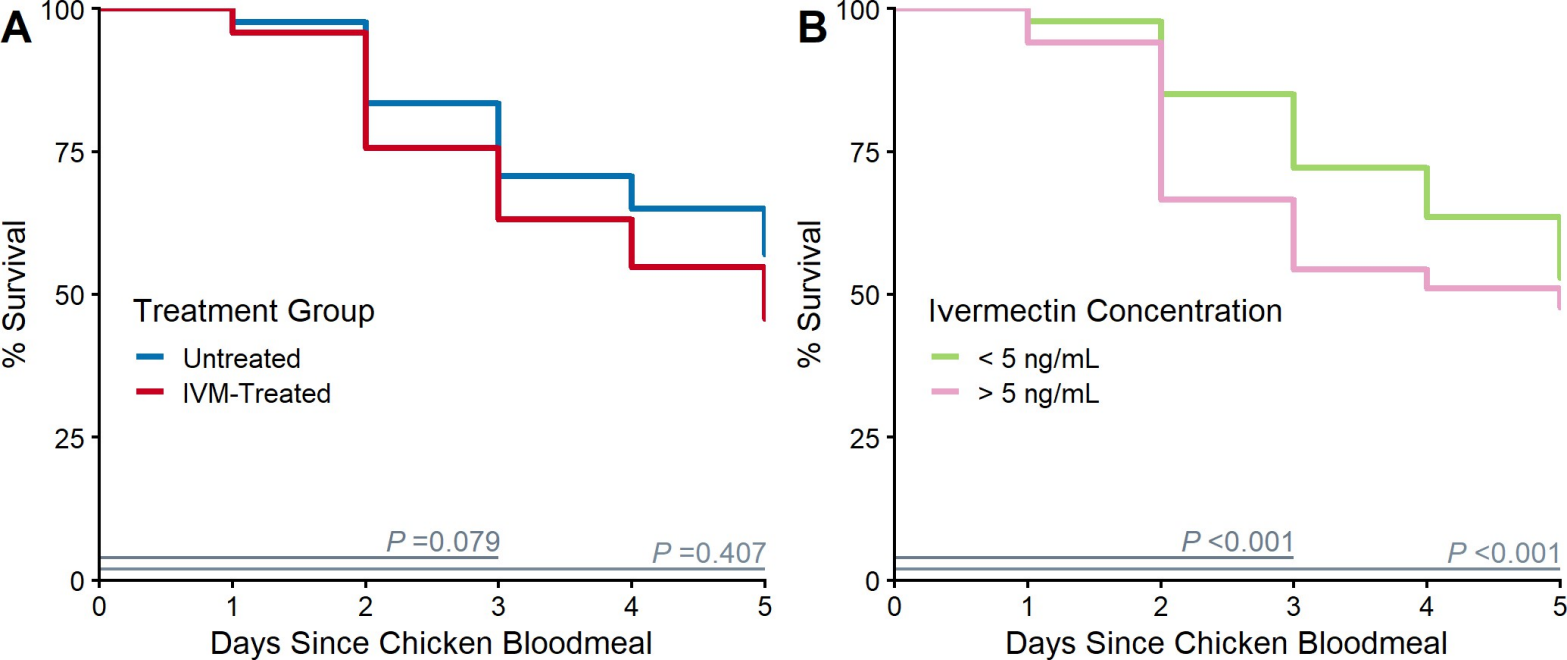
Blood feeding on ivermectin (IVM)-treated chickens increased wild *Culex tarsalis* mortality. Wild-caught *Cx*. *tarsalis* survival following blood feeding on a randomly chosen chicken from each coop A) by treatment group and B) stratified by IVM serum concentration above or below the limit of quantification (LOQ, 5 ng/mL). Mantel-Haenszel chi-square *P*-value indicated for comparison of Kaplan-Meier survival curves for 1–3 and 1–5 days (duration indicated by grey segment). IVM-related effects were expected to occur within 3 days post-bloodmeal.

Ivermectin serum concentrations (ng/mL) obtained from the four randomly selected treated chickens on the morning prior to the mosquito feeding bioassay were 32.3, 23.2, 17.0, and LOQ (5 ng/mL). During the three days post-bloodmeal when IVM-related effects were expected to occur [46], the higher mortality observed in wild *Cx*. *tarsalis* feeding on a randomly chosen IVM-treated chicken vs. an untreated chicken was approaching significance (Fig 5A, χ^2^ = 3.09, df = 1, *P* = 0.079). When stratified by the final IVM serum concentration relative to the LOQ (5 ng/mL) (Fig 5B**)**, we observed a significant difference in the mortality of wild mosquitoes feeding on a chicken with an IVM serum concentration >5 ng/mL vs. a chicken with an IVM serum concentration < 5 ng/mL during the three days post-bloodmeal (χ^2^ = 33.02, df = 1, *P <* 0.001). We observed a 45.6% morality in mosquitoes within three days post-bloodmeal on a chicken with a concentration above the LOQ. This was 16.3% greater than mortality observed during this period in mosquitoes following a bloodmeal on an untreated chicken. There was no difference in mortality between mosquitoes feeding on the chicken with a concentration at the limit of quantification vs. an untreated chicken (hazard ratio = 0.79, Z = -1.39, *P* = 0.16).

### Effect of IVM on parity of *Cx*. *tarsalis*

**Fig 6:**
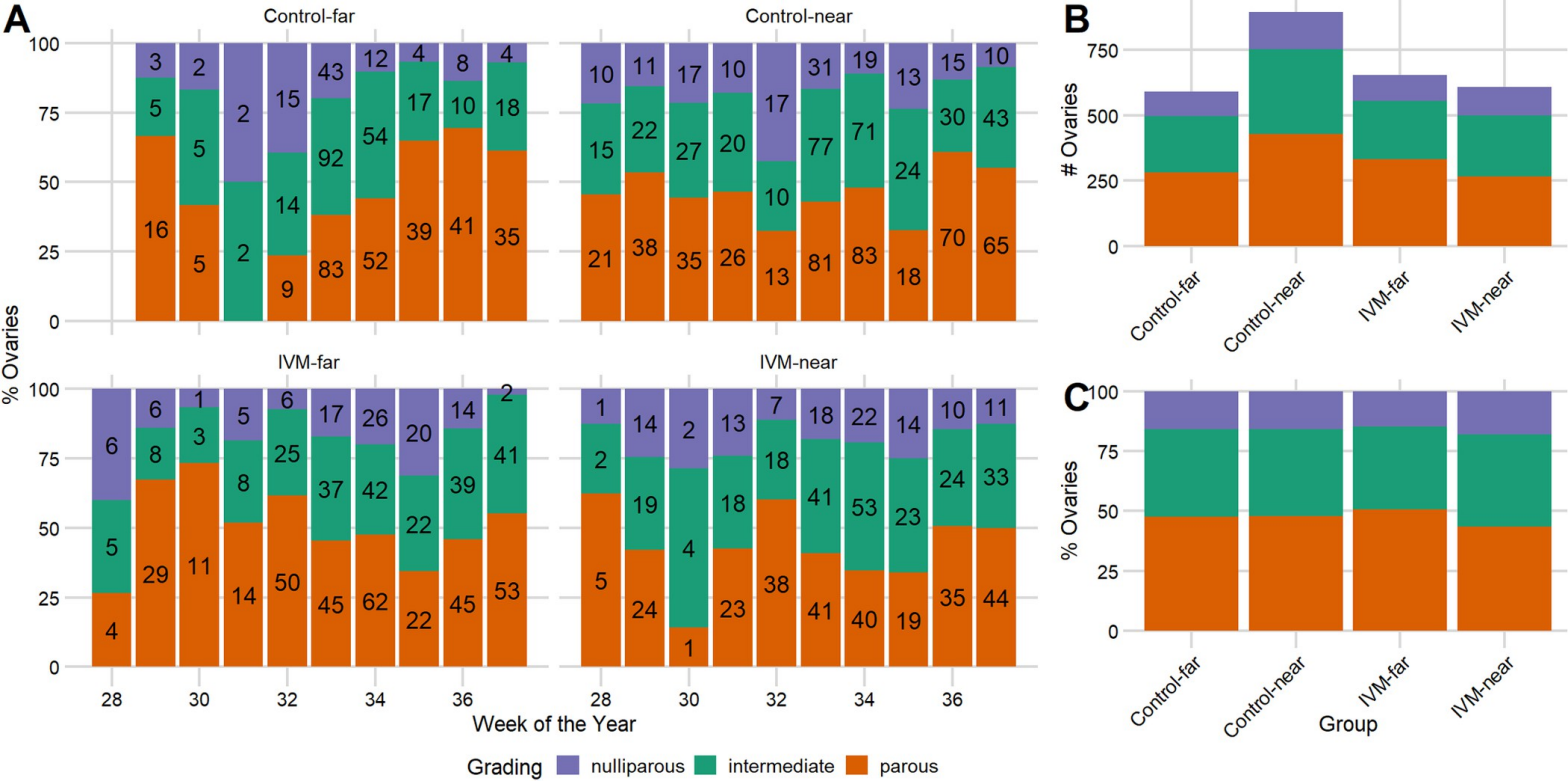
Parity rates in *Culex tarsalis* around ivermectin (IVM)-treated and untreated chicken flocks. Parity grading during the West Nile virus (WNV) season near (≤10m) and far (~150m) from treated and untreated flocks for (A) individual weeks and (B-C) collapsed by group for weeks with observations in each group (weeks 29–37). Number of ovaries in each category by week indicated in bars in A.

We observed an overall reduction in parity rates from mosquitoes collected at traps placed near vs. far from IVM-treated flocks, but parity rates varied significantly between weeks (Fig 6). Of the 3,665 total dissections, we removed 139 that contained eggs as completely developed eggs prevented visualization of the tracheoles. The nine weeks with observations in each of the four groups (i.e., weeks 29–37) encompassed 3,342 dissections. Of these, 2,748 were graded as either parous, nulliparous, or intermediate and 594 could not be evaluated due to damage to tracheoles or because ovaries were obscured by fat or reticulation. We observed a significant reduction in the overall parity rates near treated flocks compared to corresponding far sites (43.5% vs. 50.7%; χ^2^ *=* 6.225, *P* = 0.013), in contrast to nearly equal rates near and far from untreated flocks (near: 47.9%; far: 47.5%; χ^2^ *=* 0.011, *P* = 0.915) (Fig 6C).

After adjustment for the effect of weeks (i.e., seasonal variation) on parity, mosquitoes near IVM-treated flocks had reduced odds of being parous compared to mosquitoes near untreated control flocks (Table 1; odds ratio = 0.74, *P* = 0.002). However, there was no significant difference in the odds of a mosquito being parous near vs. far within each treatment group (Table 1; IVM-treated: *P* = 0.18; Control: *P* = 0.71) and parity rates at both distances of IVM-treated coops were lower than at corresponding distances from untreated control coops. See S1 Table for full model regression results, including random effects.

**Table 1:** Odds ratio (95% CI) of a mosquito being parous grouped by distances from ivermectin (IVM)-treated and untreated flocks.

Comparison	Odds Ratio	95% CI	*P-*value^
**Control-near vs. Control-far**	1.09	0.89, 1.33	0.407
**Control-near vs. IVM-near**	0.74	0.61, 0.90	0.002
**Control-near vs. IVM-far**	0.88	0.73, 1.06	0.184
**IVM-near vs IVM-far**	0.84	0.68, 1.03	0.096
**IVM-near vs. Control-far**	0.68	0.55, 0.85	< 0.001
**IVM-far vs. Control-far**	0.81	0.66, 1.00	0.046

^Wald test *P-value*

### *Cx*. *tarsalis* abundance, infection prevalence, and vector index

**Fig 7:**
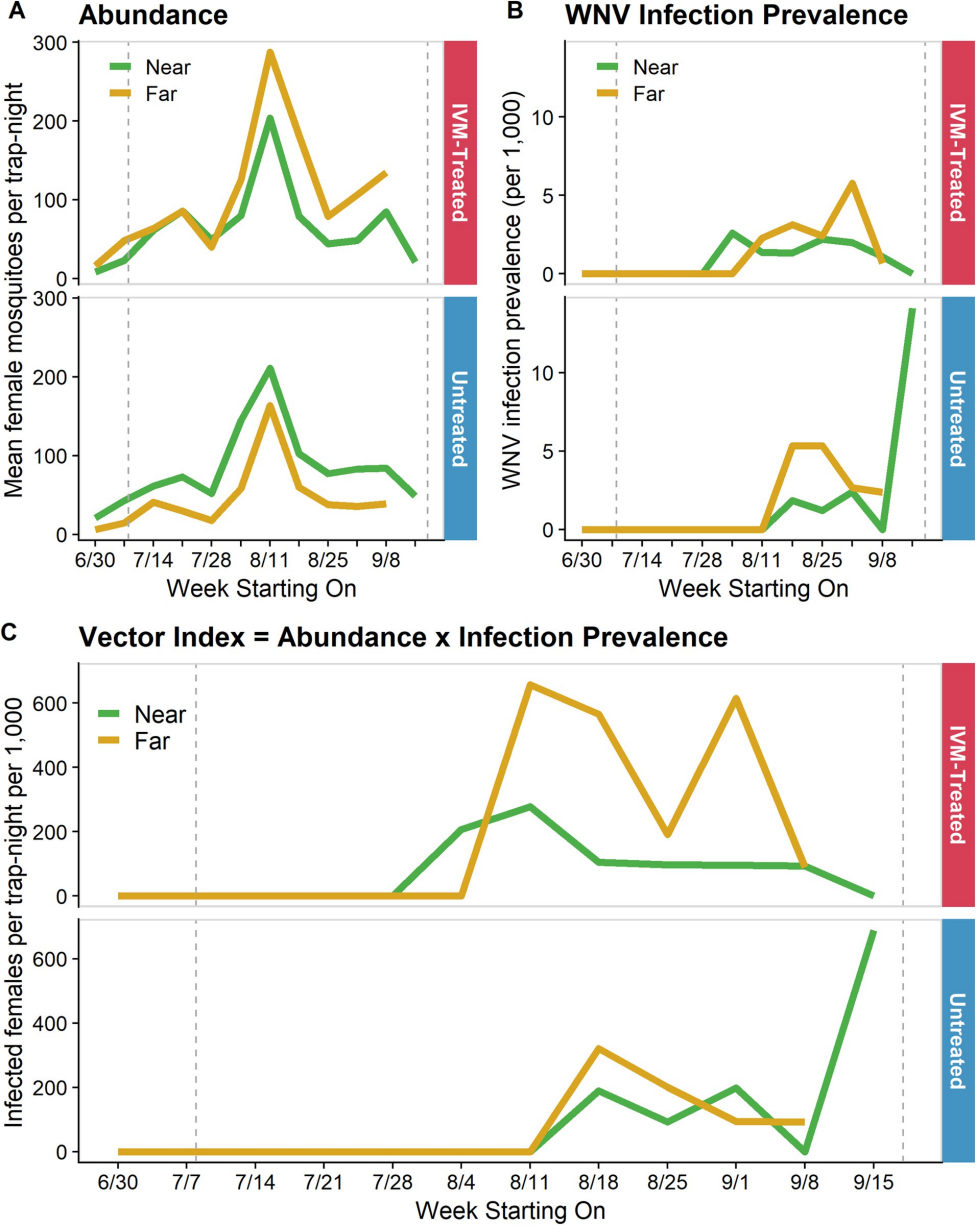
Entomological indices of *Culex tarsalis* around ivermectin (IVM)-treated and untreated chicken flocks. Weekly (A) abundance, (B) infection prevalence per 1,000, and (C) vector index (VI) near (≤ 10m) and far (~150m) from IVM-treated and untreated flocks. VI is a risk metric that approximates the number of infectious mosquitoes present as the product of abundance and infection prevalence. Vertical dashed lines indicate the first and last day IVM-treated feed was provided.

We observed trap-counts increasing sharply across all groups to a peak in early August and then decreasing into September (Fig 7A). On average 200 *Cx*. *tarsalis* were collected per trap-week. We observed a small increase in abundance in early September at both near and far distances of IVM-treated flocks. Higher average trap-counts were observed at sites closer to irrigated agriculture and in the eastern portion of Davis (S2 Fig). Excluding the final week due to small sample sizes, abundance varied significantly across weeks (F(10, 162) = 3.661, *P* < 0.001), but not across distance-treatment groups (i.e., near-treated, far-treated, near-control, far-control; F(3, 162) = 2.144, *P* = 0.097).

In terms of infection rates in mosquitoes, we observed a lower and later than average WNV season in Davis; typically, WNV is detected in late June and peaks in late August [59]. Across groups, we observed an average infection prevalence of 1.15 per 1,000 female mosquitoes tested. The initial detection of WNV occurred in early August at a near trap site around an IVM-treated flock and subsequently WNV was detected at both near and far trap sites around flocks in both treatment groups for the remainder of the study (Fig 7B). Around IVM-treated flocks, infection prevalence increased over time at far distances, peaking in early September, while remaining similar or decreasing at near distances. We observed an opposite relationship in untreated flocks where infection prevalence peaked in far traps in August and subsequently declined while infection prevalence remained similar or slightly increasing at near traps; small sample sizes in the final week resulted in a dramatic increase in estimated infection prevalence. Comparing infection prevalence across weeks with equal sample size (i.e., not the final week), there was no difference by week (F(10, 162) = 1.421, *P* = 0.175) or distance-treatment group (F(3, 162) = 2.336, *P* = 0.076).

We observed a sustained reduction in the number of infected mosquitoes, as estimated by vector index, in near vs. far traps around IVM-treated coops, while the number of infected mosquitoes was very similar at near vs. far distances from untreated locations (Fig 7C). However, there was no significant difference in vector index by week (F(10, 162) = 1.709, *P* = 0.083) or distance-treatment group (F(3, 162) = 1.589, *P* = 0.194), excluding the final week due to small sample sizes.

### Gross and histological findings in chickens

Due to time and staffing constraints, we obtained weights of chickens near the end of the study (Sept 6 and Sept 16). All chickens (*n* = 48) were weighed at the first timepoint while only 36 were weighed at the second; weights were not recorded for the remaining twelve chickens that had been necropsied on Sept 13. There was no difference in mean weight of chickens between treated and untreated flocks on either day (Sept 6: *t* = -1.472, df = 45.417, *P* = 0.148; Sept 16: *t* = -0.873, df = 33.497, *P* = 0.389). Also, there was no difference in change in weights between dates by treatment groups (*t* = 1.088, df = 33.64, *P* = 0.285).

There were no distinct pathologic findings on the postmortem examination of the IVM treated and untreated control chickens (Table 2). The main findings on gross and histological examinations were acute hepatic hemorrhage in both treated (3/6) and untreated (2/6) chickens, likely indicative of the early stages of hemorrhagic liver syndrome of unknown etiology. All birds regardless of treatment status also had mild to moderate egg yolk peritonitis which is a common condition in older laying hens [81,82]. While most birds had intestinal parasites present, one treated and one untreated chicken had no parasites observed during examination.

**Table 2:** Pathologic and histological findings at necropsy of ivermectin-treated (*n* = 6) and untreated (*n* = 6) chickens.

Tissue or Condition	Pathological or Histological Finding	Untreated	Treated	*P*-value^
Liver	Healthy	4	3	1
	Hemorrhage	2	3	
Tapeworms	None	2	1	0.318
	Few to some	4	2	
	Moderate to many	0	3	
Ascarids	None	1	5	0.080
	Few to some	4	1	
	Moderate	1	0	
Oviduct leiomyoma	Present	1	1	1
	Absent	5	5	
Lymphocytes in peripheral nerves^†^	None	0	2	0.455
	Rare to small numbers	6	4	
Lymphocytic infiltrations^‡^	Multi-systemic	6	6	1
Egg yolk peritonitis^§^	Mild to marked	6	6	1
Pneumoconiosis and/or BALT^⁋^ hyperplasia	Minimal to moderate	6	6	1

^ Fisher’s exact *P*-value

^†^ Few lymphocytes in sciatic nerves likely associated with Marek’s disease. No other significant lesions in nervous system.

^‡^ Lymphofollicular formations in heart (*n* = 7), mesentery (*n* = 12), and oviduct wall (*n* = 1) suggestive of antigenic stimulus.

^§^ Common condition in older layer hens.

^⁋^ BALT: bronchus-associated lymphoid tissue

## Discussion

Through a randomized trial in backyard chickens, we assessed the efficacy of IVM-medicated feed to suppress the abundance of WNV-infected mosquitoes and transmission of WNV. Taken together, our results suggest that IVM administration reduced WNV transmission to treated chickens and affected local *Cx*. *tarsalis* populations. Wild *Cx*. *tarsalis* had elevated mortality following a bloodmeal on chickens with IVM serum concentrations between 17–32 ng/mL, and we detected a significant reduction in mosquito parity near IVM-treated flocks. However, we did not find a difference in abundance or infection prevalence in mosquitoes between treatment groups to complete the causal chain of IVM ingestion to reduction in WNV transmission.

The estimated LC_50_ for colony *Cx*. *tarsalis* at three days post-bloodmeal from our single trial (66.03 ng/mL) was higher than previously reported (49.94 ng/mL, 95% CI: 39.71–59.93) [46], but qualitatively similar. The previous work used mosquitoes from a different colony of the same species (Bakersfield Field Station, BFS), potentially indicating a difference in susceptibility between these long-established strains. However, we used a single replicate of much smaller sample sizes than the previous work as our goal was confirmation instead of estimation so differences in magnitude should not be emphasized.

The mean IVM serum concentration observed in the treated chickens was 33.1 ng/mL, lower than the targeted level (estimated LC_50_ value), but still at mosquitocidal levels for wild *Cx*. *tarsalis*. We observed 45.6% mortality (16.3% excess mortality) in wild mosquitoes within three days of a bloodmeal on treated chickens (IVM serum concentration of 17–32 ng/mL). In the colony assay, we observed no mortality three days post-bloodmeal for mosquitoes ingesting 37.5 ng/mL, highlighting that laboratory-derived estimates may not accurately predict results in the field. While the observed mortality may only marginally impact WNV transmission, it indicates the potential for such impact. Furthermore, it is reasonable to expect that mortality would have been greater if the blood-fed wild mosquitoes were subject to natural environmental conditions rather than a protected, temperature and humidity-controlled insectary. Sublethal fitness impacts (e.g., inhibited flight, slower digestion, reduced blood-feeding frequency, and inhibited egg production) [83,84] have been documented in *Anopheles* mosquitoes. Thus, the full impact of IVM ingestion on mosquito populations and WNV transmission may be a combination of direct mortality and reduced fitness. Achieving a higher IVM serum concentration, which is the goal of future work, would be expected to result in higher daily mortality and stronger signals in entomological indices. We did not observe any difference in mosquito mortality following a bloodmeal on a treated chicken with an IVM serum concentration at the LOQ (5 ng/mL) and an untreated chicken, indicating a minimum IVM serum concentration of >5 ng/mL is required to achieve meaningful mosquitocidal effects. Serum IVM concentrations in chickens ranged widely over the study, so the exact dose ingested by biting mosquitoes at each timepoint is unclear. However, previous work indicates that even low IVM concentrations may exert strong mosquitocidal effects in the field; in a serum-replacement assay, 100% mortality in two days was observed in *Cx*. *tarsalis* (BFS) that ingested serum from a wild-caught treated grackle (5.7 ng/mL) as compared to control calf serum [46].

Previous studies in chickens indicate rapid elimination of IVM from plasma following oral treatment. Peak observed plasma concentration (10.2 ng/mL) occurred 3.36 hours after a single dose of 0.2 mg/kg of IVM administered orally via ingluvies feeding tube (IVM diluted (1:5 v/v) with propylene glycol) [85]. IVM was not detected in plasma three days post-IVM treatment. One day following a five-day IVM treatment course of 0.4 mg/kg dosing in available drinking water, a peak IVM plasma concentration of 1.07 ng/mL was observed and was no longer detected seven days after treatment ceased [86]. However, no studies assessed IVM concentrations during repeated dosing. Given the rapid elimination of IVM from plasma, treatment timing and duration are important to determine the level of IVM exposure to biting insects. Our results indicate that sustained treatment would be required to maintain mosquitocidal IVM concentrations in the blood of treated chickens. In our study, blood samples were taken in the morning around the time chickens were fed and food was often consumed before the afternoon, so IVM serum concentrations could feasibly have been lower in the evening when mosquitoes are host-seeking. Therefore, the reported IVM serum concentrations may represent the upper range of IVM to which mosquitoes were exposed. Additionally, given that these IVM serum concentrations were lower than the estimated LC_50_ values and yet we observed significant increase in mortality, achieving the laboratory-derived concentrations may not be necessary. More work is needed to understand the relationship between IVM concentration, mosquito mortality, and expected reductions in WNV transmission under natural conditions.

Successful mosquito control would be expected to cause a shift towards a lower mean age of the population due to elimination of extant adult mosquitoes that are replaced by newly emerged individuals. In this study, large sample sizes resulted in high power to detect a small difference in parity rates between groups. Parity was reduced significantly in *Cx*. *tarsalis* collected near treated flocks vs. near untreated controls, which, considered alone, suggested a possible elimination of older female mosquitoes attributable to IVM. Also, comparisons between distances within each treatment group showed that overall parity rates were lower at sites near vs. far from treated flocks, whereas rates remained nearly identical between distances for the untreated flocks. However, these distance-based comparisons were not significant for either treatment group, leaving open the possibility that some of the parity differences could have been due to chance differences in background mosquito population dynamics unrelated to treatment. Also, the observed 7.2% reduction in parity rates near vs. far from IVM-treated flocks may be insufficient to dramatically reduce WNV infection prevalence in local mosquito populations. Yet, the observed difference hints that IVM administration has the potential to cause significant enough changes in population age structure to alter WNV transmission dynamics.

Detecting a change in population age structure in natural setting following control is fraught with difficulties. Following aerial applications of adulticides, shifts in population age structure were not discernable in highly connected areas or those with high autogeny rates, but were detected in semi-isolated areas with low autogeny [35,87]. In our study, we found a relatively high parity rate (44–51%), consistent with reports of high rates of autogeny in the Sacramento Valley (54–92%) [77,78]. Highly dispersive populations of female mosquitoes of all ages could have diluted any effect of IVM on age structure. Thus, large spatial coverage and density of IVM-treated birds with higher IVM serum concentrations may likely have been required to result in dramatic differences in parity rates.

The increased mortality of wild mosquitoes feeding on treated chickens paired with the reduction in parity near treated flocks potentially supports the hypothesis that reduced seroconversions were caused by the impact of IVM on mosquito populations. Additionally, as chicken seroconversions have been shown to reflect risk for human infections [88], IVM administration could have reduced zoonotic WNV transmission risk around treated flocks.

However, we did not detect a difference in abundance or infection prevalence in *Cx*. *tarsalis* populations to fully connect the impact of IVM on mosquito populations to observed differences in WNV transmission. We observed a seasonal pattern in abundance typical of the Sacramento Valley [89,90]. Also, we found higher average trap counts at sites closer to irrigated agriculture and in the eastern portion of Davis, as previously observed [91], due to immigration of *Cx*. *tarsalis* into Davis from areas with abundant larval habitats. Continual immigration of newly emerged *Cx*. *tarsalis* from these productive larval sites likely obscured any reduction in abundance due to IVM-induced mortality; the use of IVM is unlikely to cause sustained reduction in abundance in highly connected populations. Additionally, the study period was below average for WNV infection prevalence in Davis compared to other years, which limited our ability to detect differences in mosquito infection, resulting in similar patterns across sites and treatments. Given the observed abundance and infection prevalence, we had 60% power (at an alpha of 0.05) to detect a 50% reduction in infection prevalence (i.e., 1.15/1,000 in untreated and 0.575/1,000 in treated groups). We would have needed six flocks per treatment group with six traps per flock weekly or four flocks per treatment group with 15 traps per flock weekly to achieve 80% power. This also could have contributed to an inability to identify a difference in vector index across distances and treatment groups. Because precision in estimated infection prevalence increases with trapping density [92,93], more traps may have been required to accurately estimate infection prevalence and identify differences between treatment groups.

One potential factor that could have impacted our ability to detect a difference between treatment groups was vector control. Homeowners were not given any instructions relating to personal vector control practices to encourage or discourage activities. Nonetheless, most homeowners in Davis are cognizant of the risk mosquitoes pose and likely performed general preventative measures (e.g., removing standing water on the premises). Thus, the behavior of the enrolled homeowners could be generalized to the whole city. During the study, the Sacramento-Yolo Mosquito and Vector Control District (SYMVCD) enacted both aerial (*n* = 40) and ground-based (*n* = 40) applications of insecticides in and near (within 2 km) the City of Davis to control adult mosquitoes. SYMVCD staff were blinded to treatment status of flocks and enacted control without regard to our study objectives, following their standard procedures to follow up on positive mosquito pools with vector control and proactively manage populations to prevent viral amplification. During the study period (June 15-Sept 25, 2019), there were 14 ground-applications within 500 m of treated flocks and 21 within 500 m of untreated flocks (Table A in S2 File), but this difference was not significant (Wilcoxon-Mann-Whitney Z = -0.331, asymptotic *P*-value = 0.741). The closest area treated by aerial adulticiding was ≥1,000 m from all coops. Most applications occurred later in the study period (i.e., after the Aug 13 midpoint of the study; 12 with 500m of treated coops, 20 within 500m of untreated coops), indicating a higher intensity of vector control later in the season. The higher number of sprays around untreated vs. treated coops could have reduced local mosquito indices (abundance and infection prevalence), biasing results towards null findings. However, the distribution of early vs late sprays (i.e., before and after Aug 13) was not significantly different between treatment groups (odds ratio = 3.217, Fisher exact *P*-value = 0.551; Table B in S2 File). This factor should be considered in future work to assess the impact of vector control applications on local mosquito populations.

We did not detect any negative health effects that were attributable to the sustained IVM treatment in chickens; typical IVM dosing schemes for parasites in birds involve a limited number and duration of treatment (1–2 doses over 7–14 days) [53,54]. Previously reported side effects of IVM toxicity in birds include slight somnolence, listlessness, ataxia, and death [52]. None of these were observed during daily checks. All birds, regardless of treatment status, had evidence of initial stages of hemorrhagic liver syndrome, a condition that is very frequent in backyard chickens and most common in the summer months (July-August) in the study region [94]. All birds also had yolk peritonitis, which was an incidental finding that is common in older laying hens of the age used in this study [81,82].

Ten of twelve birds had relatively low numbers of intestinal cestodes and ascarids. While oral, subcutaneous, and intramuscular administrations of IVM are used to treat nematode infestations in birds, including chickens [54], we did not observe a significant reduction in ascarid loads at necropsy (*P* = 0.08), potentially due to IVM serum concentrations achieved, small sample sizes, or qualitative measures of loads used. Similarly, a previous study found that while highly effective against reducing experimental *Ascaridia galli* infections in chickens (89.8–95% reduction), two subcutaneous ivermectin doses of 0.3 mg/kg two weeks apart were not totally effective in eliminating the parasite [53]. Thus, while a side benefit of IVM treatment may be a reduction, complete elimination of any nematode loads may not be expected.

Our decision to randomize treatment status among the eight flock sites resulted in some limitations of our statistical power to detect treatment effects. Abundance and WNV infection rate in *Cx*. *tarsalis* exhibited clear spatial patterns from east to west across the city of Davis during the study period, as reported previously [91]. Therefore, blocking flocks spatially into treated and untreated pairs to ensure equal representation of treatment groups across this gradient in entomological indices might have increased our statistical power.

The unexpected number of seropositive chickens at the start of our study resulted in smaller than expected and unequal group sizes between treatment status, potentially reducing our power to detect differences. The chickens had been housed outdoors during the previous year, but we had not expected many seropositive chickens based on low reported annual seroconversions in previous sentinel flocks in Davis [95]. Despite the resulting reductions in treated flock sizes, we were able to detect a significant difference in seroconversion rates between groups, suggesting that chickens are a more sensitive indicator of WNV transmission than mosquito collections [92]. Future studies employing a larger sample size of spatially paired flock sites would be needed to further support these findings.

Homeowners reported that they enjoyed hosting the chicken flocks for this study, but this led to the unintended consequence that some occasionally supplemented our study diet with food scraps, contrary to our instructions. We observed five instances of unquantifiable IVM concentrations in chickens in one of our treated coops, presumably due to the supplemental provision of alternative food sources. While all feed and powdered IVM was ingested each day, some individual chickens may have refrained from eating our provided food, preferring the supplemental items, and thus stopped self-medicating for a period, resulting in some degree of IVM washout and variability among chickens. We observed evidence of supplemental feeding in other flocks as well, including other treated flocks, thus reducing the IVM serum concentrations in treated chickens and potentially biasing our results towards no effect of IVM on mosquito populations. We did not track the timing or identity of items provided for supplemental feeding, so we were unable to confirm supplemental feeding was the cause of this observation or quantify the potential extent of the impact on our findings. Even when supplemental food sources were present, no food or IVM powder residues remained in the feeding dishes between days, thus indicating that there was not a significant difference in palatability of IVM-treated vs. untreated feed.

The potential impact of supplemental, untreated food sources on this study’s findings is of relevance for transitioning the strategy to wild birds. The myriad of alternative food sources available to wild birds, in addition to IVM-treated feed, could similarly result in highly variable IVM concentrations across the season and between individual birds. Future work is needed to understand birdfeeder usage patterns and to develop a feed formulation with extended-release properties to maintain stable IVM serum concentrations; our use of powdered IVM serves as an initial baseline ahead of further work on feed formulations to achieve appropriate targets for dosing and half-life in wild birds.

Similar to previous evaluations using the tracheation method to age-grade mosquitoes (8–25%) [74,75], a portion of our ovarian slide mounts (19.2% overall and 17.8% in weeks 29–37) were unsatisfactory for classification due to obscuring dark masses likely attributable to egg yolk protein, loss of tracheation during dissection, or presence of debris and fat occluding the tracheoles. Despite this, we did detect a difference in parity, but the number of ungradable mounts prolonged the processing time required. This high level of ungradable specimens across studies highlights the need for improved age-grading techniques. An alternative method, outlined by Polovodova [96] and applied to *Cx*. *tarsalis* by Nelson [97], uses dilatation in follicular tubes following development and deposition of an egg batch to successfully differentiates nulliparous and parous *Cx*. *tarsalis* females, removing the ambiguity of intermediate classifications resulting from the tracheation method [76]. However, this method still requires dissection and a relatively long processing time per mosquito [98]. A recent modification to a method suggested by Perry [99] based on wing wear uses the number of scales along the distal edge of wings of *Anopheles gambiae* to determine the relative age and can be automated [100]. If a similar relationship of scale loss and age holds for *Cx*. *tarsalis* populations, this automatable method would provide rapid and fine-scale resolution to relative age, but loss of scales due to passage through the fan and time spent in the collection container may still result in inappropriate grading of trap collected mosquitoes.

Use of backyard chickens as the means for exposing wild mosquitoes to IVM was intended as a first step toward potential future uses of IVM in backyard bird feeders as a way of achieving targeted WNV control near human residences. Compared to studies with wild birds, chickens had the advantage of remaining in a single location, and they are fed upon frequently by *Cx*. *tarsalis* where they are present [55,56]. We anticipated that these factors would give the greatest chance at identifying IVM’s spatial effects on mosquitoes and WNV transmission. Due to limiting factors like lower IVM serum concentrations in chickens, lower-than-average infection prevalence in mosquitoes, and high rates of immigration of mosquitoes, we were only able to detect partial support for these conclusions. For long-term considerations, IVM, a lipophilic drug [101], is known to accumulate in eggs [86,102], which is almost certain to limit appeal of a chicken-based control strategy among homeowners who typically eat the chickens’ eggs. The FDA has not set an IVM tolerance for eggs, and homeowners were advised not to ingest eggs from treated chickens during this study. Assessing IVM concentrations in chicken tissues and eggs over time, and the human health ramifications of ingesting these edible products was outside the scope of this study. Assessment of IVM concentrations in eggs should be performed in the future if eggs are to be consumed by humans. Additionally, the impact of IVM on embryo development and hatching success needs investigation. We are not aware of any studies assessing this in birds and it is vital to ascertain prior to deployment of this strategy.

Another avenue warranting further investigation is the potential development of IVM resistance in *Culex* mosquitoes. Repeated mass drug administration (MDA) of IVM to entire flocks or herds of livestock has led to resistance in *Haemonchus contortus*, a common gastrointestinal roundworm in sheep, cattle, goats, and horses [103,104]. There is also evidence of IVM resistance in the canine heartworm, *Dirofilaria immitis* [105]. In arthropods, resistance to ivermectin has been reported in *Rhipicephalus microplus*, the Australian cattle tick [106]. This tick is a one-host tick, meaning all life stages feed only on a single host species, thus making it more sensitive to development of resistance than multi-host ticks. In terms of mosquito populations, a lab study identified mutations in cytochrome P450 enzymes as a potential pathway to the development of resistance in *Anopheles gambiae* [107]. Additionally, there is the potential for the development of cross-resistance between IVM and other pesticides that share the same targets (GABA receptors; [108]) or through the same transporter proteins (modulators of P-glycoproteins; [109]). This is an area of high concern for the efficacy of MDA in humans to control tropical diseases like malaria, lymphatic filariasis, and onchocerciasis. However, there is no evidence from the field of the development of such resistance in mosquito populations [45,49]. Care needs to be taken to minimize the potential for resistance. In MDAs and our proposed treatment of birds, segments of the population would remain untreated, allowing for refugia where selection pressures from IVM are absent, thus minimizing selection potential for resistance.

In conclusion, following oral administration of IVM medicated feed to backyard chickens, we detected evidence of a reduction in WNV transmission to chickens, accompanied by mixed findings regarding the entomological impact of IVM on *Culex* mosquito populations. While not conclusively linked to IVM administration, this study adds support for the plausibility of a causal relationship between IVM treatment of birds and reduced WNV transmission. We observed fewer WNV seroconversions in treated chickens than untreated chickens, a reduction in parity rates of *Cx*. *tarsalis* near treated vs. untreated flocks, and increased mortality in wild mosquitoes following a bloodmeal on treated chickens vs. untreated chickens. Ivermectin serum concentrations resulting in increased mortality ranged between 17–32 ng/mL, while a concentration at the limit of quantification (5 ng/mL) did not increase mortality compared to untreated chickens, indicating that a certain threshold may be required to cause significant mosquitocidal impacts. IVM serum concentrations varied widely across the season with a mean of 33.1 ng/mL (range: <5–155.2 ng/mL), lower than the estimated LC50 (37.5 ng/mL). We did not observe a difference in either abundance or WNV infection prevalence in *Cx*. *tarsalis* populations between treated and untreated sites, potentially due to sustained immigration of newly emerged individuals, lower-than-average WNV activity in the study area, and below target IVM serum concentrations in chickens. Also, more traps may have been required to accurately estimate infection prevalence and identify differences between treatment groups. Sustained oral ingestion of IVM did not result in any adverse events, highlighting the safety of this method. Future work aims to transition to wild birds and develop a commercial treated birdfeed for homeowner use to reduce WNV risk on the local neighborhood scale.

## Supporting information

S1 Fig *Culex tarsalis* survival in bioassay with ivermectin (IVM). *Cx*. *tarsalis* (Kern Natural Wildlife Reserve colony) survival following a membrane bloodmeal containing serial dilutions of IVM. Number of blood-fed female mosquitoes at each IVM concentration indicated.
(TIF)

S2 Fig Entomological indices of *Culex tarsalis* by study site and treatment status. Weekly (A) abundance, (B) infection prevalence per 1,000, and (C) vector index (VI) near (≤ 10m) and far (~150m) from ivermectin (IVM)-treated and untreated flocks. VI is a risk metric that approximates the number of infectious mosquitoes present as the product of abundance and infection rate. Individual plot headers indicate site number (see Fig 1) and treatment status and are ordered by spatial location west to east (L to R).
(TIF)

S1 Table Final model estimates. Fixed and random effect estimates from mixed effects logistic regression for parity in *Cx*. *tarsalis* mosquitoes at near and far distances from ivermectin (IVM)-treated and untreated control flocks.
(DOCX)

S1 Dataset Data from colony mosquito membrane feeding assay. Daily number of surviving *Cx*. *tarsalis* (Kern National Wildlife Reserve (KNWR) colony) following an artificial bloodmeal with serial dilutions of ivermectin (IVM; ng/mL). NA: not assessed.
(CSV)

S2 Dataset Data from testing blood samples for West Nile virus antibodies. Date of blood sample collection and final test result for presence of IgG antibodies.
(CSV)

S3 Dataset Data of ivermectin serum concentrations (ng/mL) in chickens. Quantification of ivermectin in serum samples using high-performance liquid chromatography.
(CSV)

S4 Dataset Data from field-caught mosquito bioassay on study chickens. Daily number of field-caught surviving *Cx*. *tarsalis* following a bloodmeal on a randomly selected chicken from each flock. Serum ivermectin (IVM; ng/mL) indicated. LOQ: limit of quantification (5 ng/mL).
(CSV)

S5 Dataset Data from parity gradings of *Cx*. *tarsalis* using ovarian tracheation techniques. Grading of ovarian dissections.
(CSV)

S6 Dataset Data of mosquito collections in Davis, California. Species and sex of mosquitoes collected in CO_2_-baited mosquito traps during June-September 2019.
(CSV)

S7 Dataset Data of test results of mosquito pools. Each pool screened with multiplex RT-PCR for West Nile virus (WNV), western equine encephalomyelitis virus (WEEV), and St. Louis encephalitis virus (SLEV).
(CSV)

S8 Dataset Data of chicken weights (kg). Weights obtained with spring scales. Chickens randomly selected for necropsy were not present to be weighed at the second timepoint.
(CSV)

S1 File Data dictionary for data presented in S1–S8 Datasets. Description of variables with indicated datasets in which each variable is present.
(CSV)

S2 File Organized ground and aerial-based insecticide applications during study period. Applications occurred June 15-Sept 25, 2019 by Sacramento-Yolo Mosquito and Vector Control District within 2 km of the city limits of Davis, California. **Table A:** Total ground-based spray events within 500m of each coop (June 15-Sept 25). **Table B:** Ground-based sprays within 500m of coops, occurring early and late in the study period.
(DOCX)

## References

[1] Hayes EB, Sejvar JJ, Zaki SR, Lanciotti RS, Bode A V, Campbell GL. Virology, pathology, and clinical manifestations of West Nile virus disease. Emerg Infect Dis. 2005;11(8):1174–9. doi: 10.3201/eid1108.050289b 16102303 PMC3320472

[2] McLean RG, Ubico SR, Docherty DE, Hansen WR, Sileo L, McNamara TS. West Nile virus transmission and ecology in birds. Ann N Y Acad Sci. 2001;951:54–7. doi: 10.1111/j.1749-6632.2001.tb02684.x 11797804

[3] Kilpatrick AM, LaDeau SL, Marra PP. Ecology of West Nile virus transmission and its impact on birds in the western hemisphere. Auk. 2007;124(4):1121–36.

[4] Turell MJ, Dohm DJ, Sardelis MR, O Guinn ML, Andreadis TG, Blow JA. An update on the potential of North American mosquitoes (Diptera: Culicidae) to transmit West Nile virus. J Med Entomol. 2005;42(1):57–62. F doi: 10.1093/jmedent/42.1.57 15691009

[5] Kramer LD, Styer LM, Ebel GD. A global perspective on the epidemiology of West Nile virus. Annu Rev Entomol. 2008;53:61–81. doi: 10.1146/annurev.ento.53.103106.093258 17645411

[6] Karabatsos N. International catalogue of arboviruses: including certain other viruses of vertebrates. 3rd ed. San Antonio, Texas: American Society of Tropical Medicine and Hygiene Subcommittee on Information Exchange of the American Committee on Arthropod-borne Viruses; 1985.

[7] Rosenberg R, Lindsey NP, Fischer M, Gregory CJ, Hinckley AF, Mead PS, et al. Vital signs: trends in reported vectorborne disease cases—United States and territories, 2004–2016. Morb Mortal Wkly Rep. 2018;67(17):496–501. doi: 10.15585/mmwr.mm6717e1 29723166 PMC5933869

[8] Mostashari F, Bunning ML, Kitsutani PT, Singer DA, Nash D, Cooper MJ, et al. Epidemic West Nile encephalitis, New York, 1999: Results of a household-based seroepidemiological survey. Lancet. 2001;358(9278):261–4. doi: 10.1016/S0140-6736(01)05480-0 11498211

[9] Hughes JM, Wilson ME, Sejvar JJ. The long-term outcomes of human West Nile virus infection. Clin Infect Dis. 2007;44(12):1617–24. doi: 10.1086/518281 17516407

[10] Centers of Disease Control and Prevention. West Nile virus disease cases and deaths reported to CDC by year and clinical presentation, 1999–2019 [Internet]. Final cumulative maps & data for 1999–2019. 2020 [cited 2021 Jan 3]. Available from: https://www.cdc.gov/westnile/statsmaps/cumMapsData.html#three

[11] Ronca SE, Murray KO, Nolan MS. Cumulative incidence of West Nile virus infection, continental United States, 1999–2016. Emerg Infect Dis. 2019;25(2):325–7. doi: 10.3201/eid2502.180765 30666940 PMC6346444

[12] Centers for Disease Control and Prevention. Average annual incidence of West Nile virus neuroinvasive disease reported to CDC by county, 1999–2018 [Internet]. Final cumulative maps & data for 1999–2018. 2020. Available from: https://www.cdc.gov/westnile/statsmaps/cumMapsData.html

[13] Rochlin I, Faraji A, Healy K, Andreadis TG. West Nile virus mosquito vectors in North America. J Med Entomol. 2019;1–16. doi: 10.1093/jme/tjy197 31549725

[14] Kovach TJ, Kilpatrick AM. Increased human incidence of West Nile virus disease near rice fields in California but not in southern United States. Am J Trop Med Hyg. 2018;99(1):222–8. doi: 10.4269/ajtmh.18-0120 29714160 PMC6085780

[15] Eisen L, Barker CM, Moore CG, Pape WJ, Winters AM, Cheronis N. Irrigated agriculture is an important risk factor for West Nile virus disease in the hyperendemic Larimer-Boulder-Weld area of North Central Colorado. J Med Entomol. 2010;47(5):939–51. doi: 10.1603/me10036 20939393

[16] Gubler DJ, Campbell GL, Nasci R, Komar N, Petersen L, Roehrig JT. West Nile virus in the United States: guidelines for detection, prevention, and control. Viral Immunol. 2000;13(4):469–75. doi: 10.1089/vim.2000.13.469 11192293

[17] California Department of Public Health, Mosquito and Vector Control Association of California, University of California. California mosquito-borne virus surveillance & response plan. 2020; https://westnile.ca.gov/resources_reports.php?resource_category_id=9

[18] Rose RI. Pesticides and public health: integrated methods of mosquito management. Emerg Infect Dis. 2001;7(1):17–23. doi: 10.3201/eid0701.010103 11266290 PMC2631680

[19] Anderson JF, Ferrandino FJ, Dingman DW, Main AJ, Andreadis TG, Becnel JJ. Control of mosquitoes in catch basins in Connecticut with *Bacillus thuringiensis israelensis*, *Bacillus sphaericus*, and spinosad. J Am Mosq Control Assoc. 2011;27(1):45–55. doi: 10.2987/10-6079.1 21476447

[20] Stockwell PJ, Wessell N, Reed DR, Kronenwetter-Koepel TA, Reed KD, Turchi TR, et al. A field evaluation of four larval mosquito control methods in urban catch basins. J Am Mosq Control Assoc. 2006;22(4):666–71. doi: 10.2987/8756-971X(2006)22[666:AFEOFL]2.0.CO;2 17304935

[21] Harbison JE, Nasci R, Runde A, Henry M, Binnall J, Hulsebosch B, et al. Standardized operational evaluations of catch basin larvicides from seven mosquito control programs in the Midwestern United States during 2017. J Am Mosq Control Assoc. 2018;34(2):107–16. doi: 10.2987/18-6732.1 31442163

[22] Harbison JE, Corcoran PC, Runde A, Henry M, Xamplas C, Nasci RS. Variable efficacy of extended-release mosquito larvicides observed in catch basins in the Northeast Chicago Metropolitan Area. Environ Health Insights. 2016;10:2014–7. doi: 10.4137/EHI.S38096 27103818 PMC4833430

[23] McMillan JR, Blakney RA, Mead DG, Coker SM, Morran LT, Waller LA, et al. Larviciding *Culex* spp. (Diptera: Culicidae) populations in catch basins and its impact on West Nile virus transmission in urban parks in Atlanta, GA. J Med Entomol. 2019;56(1):222–32. doi: 10.1093/jme/tjy174 30295776

[24] Lothrop HD, Lothrop BB, Gomsi DE, Reisen WK. Intensive early season adulticide applications decrease arbovirus transmission throughout the Coachella Valley, Riverside County, California. Vector Borne Zoonotic Dis. 2008;8(4):475–89. doi: 10.1089/vbz.2007.0238 18494603 PMC2978539

[25] Lothrop HD, Lothrop B, Palmer M, Wheeler S, Gutierrez A, Miller P, et al. Evaluation of pyrethrin aerial ultra-low volumet applications for adult *Culex tarsalis* control in the desert environments of the Coachella Valley, Riverside county, California. J Am Mosq Control Assoc. 2007;23(4):405–19. doi: 10.2987/5623.1 18240517

[26] Mutebi JP, Delorey MJ, Jones RC, Plate DK, Gerber SI, Gibbs KP, et al. The impact of adulticide applications on mosquito density in Chicago, 2005. J Am Mosq Control Assoc. 2011;27(1):69–76. doi: 10.2987/10-6045.1 21476450

[27] Reddy MR, Spielman A, Lepore TJ, Henley D, Kiszewski AE, Reiter P. Efficacy of resmethrin aerosols applied from the road for suppressing *Culex* vectors of West Nile virus. Vector-Borne Zoonotic Dis. 2006;6(2):117–27. doi: 10.1089/vbz.2006.6.117 16796509

[28] Holcomb KM, Reiner RC, Baker CM. Spatio-temporal impacts of aerial adulticide applications on populations of West Nile virus vector mosquitoes. Parasit Vectors. 2021;14(120):1–15. doi: 10.1186/s13071-021-04616-6 33627165 PMC7905633

[29] Mount GA, Biery TL, Haile DG. A review of ultralow-volume aerial sprays of insecticide for mosquito control. J Am Mosq Control Assoc. 1996;12(4):601–18. 9046465

[30] Macedo PA, Schleier JJ, Reed M, Kelley K, Goodman GW, Brown DA, et al. Evaulation of efficacy and human health risk of aerial ultra-low volume applications of pyrethrins and piperonyl butoxide for adult mosquito management in response to West Nile virus activity in Sacramento county, California. J Am Mosq Control Assoc. 2010;26(1):57–66. doi: 10.2987/09-5961.1 20402352

[31] Elnaiem DA, Kelley K, Wright S, Laffey R, Yoshimura G, Reed M, et al. Impact of aerial spraying of pyrethrin insecticide on *Culex pipiens* and *Culex tarsalis* (Diptera: Culicidae) abundance and West Nile virus infection rates in an urban/suburban area of Sacramento County, California. J Med Entomol. 2008;45(4):751–7. doi: 10.1603/0022-2585(2008)45[751:ioasop]2.0.co;2 18714879

[32] Palmisano CT, Taylor V, Caillouet K, Byrd B, Wesson DM. Impact of West Nile virus outbreaks upon St. Tammany parish mosquito abatement district. J Am Mosq Control Assoc. 2005;21(1):33–8. doi: 10.2987/8756-971X(2005)21[33:IOWNVO]2.0.CO;2 15825759

[33] Carney RM, Husted S, Jean C, Glaser C, Kramer V. Efficacy of aerial spraying of mosquito adulticide in reducing incidence of West Nile Virus, California, 2005. Emerg Infect Dis. 2008;14(5):747–54. doi: 10.3201/eid1405.071347 18439356 PMC2600250

[34] Barber LM, Schleier JJ, Peterson RKD. Economic cost analysis of West Nile virus outbreak, Sacramento County, California, USA, 2005. Emerg Infect Dis. 2010;16(3):480–6. doi: 10.3201/eid1603.090667 20202424 PMC3322011

[35] Reisen WK, Yoshimura G, Reeves WC, Milby MM, Meyer RP. The impact of aerial applications of ultra-low volume adulticides on *Culex tarsalis* populations (Diptera: Culicidae) in Kern County, California, USA, 1982. J Med Entomol. 1984;21(5):573–85. doi: 10.1093/jmedent/21.5.573 6209396

[36] Nielsen CF, Reisen WK, Armijos V, Wheeler S, Kelley K, Brown D. Impact of climate variation and adult mosquito control on the West Nile virus epidemic in Davis, California during 2006. Proc Pap Mosq Vector Control Assoc Calif. 2007;75:125–30.

[37] Iyaniwura TT. Non-target and environmental hazards of pesticides. Rev Environ Health. 1991;9(3):161–76. doi: 10.1515/reveh.1991.9.3.161 1792388

[38] Bonds JA. Ultra-low-volume space sprays in mosquito control: a critical review. Med Vet Entomol. 2012;26(2):121–30. doi: 10.1111/j.1365-2915.2011.00992.x 22235908

[39] Oberhauser KS, Manweiler SA, Lelich R, Blank M, Batalden R V., De Anda A. Impacts of ultra-low volume resmethrin applications on non-target insects. J Am Mosq Control Assoc. 2009;25(1):83–93. doi: 10.2987/08-5788.1 19432072

[40] Rasmussen JJ, Wiberg-Larsen P, Kristensen EA, Cedergreen N, Friberg N. Pyrethroid effects on freshwater invertebrates: A meta-analysis of pulse exposures. Environ Pollut. 2013;182:479–85. doi: 10.1016/j.envpol.2013.08.012 24035458

[41] Thier A. Balancing the risks: Vector control and pesticide use in response to emerging illness. J Urban Heal Bull New York Acad Med. 2001;78(2):372–81. doi: 10.1093/jurban/78.2.372 11419588 PMC3456351

[42] Roberts DR, Andre A. Insecticide resistance issues in vector-borne disease control. Am J Trop Med Hyg. 1994;50(6):21–34. doi: 10.4269/ajtmh.1994.50.21 8024082

[43] Liu NN. Insecticide resistance in mosquitoes: Impact, mechanisms, and research directions. Annu Rev Entomol Vol 60. 2015;60:537–59. 10.1146/annurev-ento-010814-020828 25564745

[44] Watts G. Nobel awarded to discoverers of ivermectin and artemisinin. BMJ. 2015;351:h5352. doi: 10.1136/bmj.h5352 26443616

[45] Laing R, Gillan V, Devaney E. Ivermectin–old drug, new tricks? Trends Parasitol. 2017;33(6):463–72. doi: 10.1016/j.pt.2017.02.004 28285851 PMC5446326

[46] Nguyen C, Gray M, Burton TA, Foy SL, Foster JR, Gendernalik AL, et al. Evaluation of a novel West Nile virus transmission control strategy that targets *Culex tarsalis* with endectocide-containing blood meals. PLoS Negl Trop Dis. 2019;13(3):e0007210. doi: 10.1371/journal.pntd.0007210 30845250 PMC6424467

[47] Sylla M, Kobylinski KC, Gray M, Chapman PL, Sarr MD, Rasgon JL, et al. Mass drug administration of ivermectin in south-eastern Senegal reduces the survivorship of wild-caught, blood fed malaria vectors. Malar J. 2010;9:365. doi: 10.1186/1475-2875-9-365 21171970 PMC3016374

[48] Kobylinski KC, Sylla M, Chapman PL, Sarr MD, Foy BD. Ivermectin mass drug administration to humans disrupts malaria parasite transmission in Senegalese villages. Am J Trop Med Hyg. 2011;85(1):3–5. doi: 10.4269/ajtmh.2011.11-0160 21734116 PMC3122335

[49] Alout H, Foy BD. Ivermectin: a complimentary weapon against the spread of malaria? Expert Rev Anti Infect Ther. 2017;15(3):231–40. doi: 10.1080/14787210.2017.1271713 27960597 PMC5538249

[50] 103d Congress. American Medicinal Drug Use Clarification Act of 1994. 108 United States of America; 1994 p. 4153–5.

[51] Lierz M. Evaluation of the dosage of ivermectin in falcons. Vet Rec. 2001;148(19):596–600. doi: 10.1136/vr.148.19.596 11386446

[52] Zeman P. Systemic efficacy of ivermectin against *Dermanyssus gallinae* (De Geer, 1778) in fowls. Vet Parasitol. 1987;23:141–6. doi: 10.1016/0304-4017(87)90032-x 3564341

[53] Sharma RL, Bhat TK, Hemaprasanth. Anthelmintic activity of ivermectin against experimental *Ascaridia galli* infection in chickens. Vet Parasitol. 1990;37:307–14. doi: 10.1016/0304-4017(90)90014-3 2267731

[54] Clyde VL, Patton S. Diagnosis, treatment, and control of common parasites in companion and aviary birds. Semin Avian Exot Pet Med. 1996;5(2):75–84.

[55] Thiemann TC, Lemenager DA, Kluh S, Carroll BD, Lothrop HD, Reisen WK. Spatial variation in host feeding patterns of *Culex tarsalis* and the *Culex pipiens* complex (Diptera: Culicidae) in California. J Med Entomol. 2012;49(4):903–16. doi: 10.1603/me11272 22897051 PMC3542768

[56] Tempelis CH, Reeves WC, Bellamy RE, Lofy MF. A three-year study of the feeding habits of *Culex tarsalis* in Kern County, California. Am J Trop Med Hyg. 1965;14:170–7. doi: 10.4269/ajtmh.1965.14.170 14248992

[57] Langevin SA, Bunning M, Davis B, Komar N. Experimental infection of chickens as candidate sentinels for West Nile virus. Emerg Infect Dis. 2001;7(4):726–9. doi: 10.3201/eid0704.010422 11585538 PMC2631771

[58] R Core Team. R: A language and environment for statistical computing. Vienna, Austria: R Foundation for Statisitcal Computing; 2020. Available from: https://www.r-project.org/.

[59] California Vectorborne Disease Surveillance System (CalSurv). 2018. Available from: http://vectorsurv.org.

[60] Hayes R, Bennett S. Simple sample size calculations for cluster-randomized trials. Int J Epidemiol. 1999;28:319–26. doi: 10.1093/ije/28.2.319 10342698

[61] Patiris PJ, Oceguera LF, Peck GW, Chiles RE, Reisen WK, Hanson C V. Serologic diagnosis of West Nile and St. Louis encephalitis virus infections in domestic chickens. Am J Trop Med Hyg. 2008 Mar;78(3):434–41. 18337340

[62] Taketa-Graham M, Powell Pereira JL, Baylis E, Cossen C, Oceguera L, Patiris P, et al. Short report: High throughput quantitative colorimetric microneutralization assay for the confirmation and differentiation of West Nile virus and St. Louis encephalitis virus. Am J Trop Med Hyg. 2010;82(3):501–4. doi: 10.4269/ajtmh.2010.09-0297 20207881 PMC2829917

[63] Therneau TM. A package for survival analysis in R. 2020. version 3.1–12. Available from: https://cran.r-project.org/package=survival.

[64] Berendsen BJA, Mulder PPJ, van Rhijn H (J) A. The derivatisation of avermectins and milbemycins in milk: New insights and improvement of the procedure. Anal Chim Acta. 2007;585(1):126–33. doi: 10.1016/j.aca.2006.12.013 17386656

[65] Prieto JG, Merino G, Pulido MM, Estevez E, Molina AJ, Vila L, et al. Improved LC method to determine ivermectin in plasma. J Pharm Biomed Anal. 2003;31(4):639–45. doi: 10.1016/s0731-7085(02)00720-3 12644190

[66] Kramer LD, Presser SB, Houk EJ, Hardy JL. Effect of the anesthetizing agent triethylamine on western equine encephalomyelitis and St. Louis encephalitis viral titers in mosquitoes (Diptera: Culicidae). J Med Entomol. 1990;27(6):1008–10. doi: 10.1093/jmedent/27.6.1008 2280383

[67] Mosquito and Vector Control Association of California. Identification of the mosquitoes of California. Elk Grove, California: Mosquito and Vector Control Association of California (MVCAC); 1998.

[68] California Department of Public Health. Mosquito pool and sentinel chicken testing. 2021; Available from: https://westnile.ca.gov/resources_reports.php?resource_category_id=12.

[69] Brault AC, Fang Y, Reisen WK. Multiplex qRT-PCR for the detection of western equine encephalomyelitis, St. Louis encephalitis, and West Nile viral RNA in mosquito pools (Diptera: Culicidae). J Med Entomol. 2015;52(3):491–9. doi: 10.1093/jme/tjv021 26334826 PMC4581483

[70] Gujral IB, Zielinski-Gutierrez EC, LeBailly A, Nasci R. Behavioral risks for West Nile Virus disease, northern Colorado, 2003. Emerg Infect Dis. 2007;13(3):419–25. doi: 10.3201/eid1303.060941 17552095 PMC2725886

[71] Fox J, Weisberg S. An R companion to applied regression. Third Edit. Thousand Oaks, CA: Sage; 2019.

[72] Detinova TS, Beklemishev WN, Bertram DS. Age-grading methods in Diptera of medical importance with special reference to some vectors of malaria. Geneva; 1962. 13885800

[73] Meadows KE. A simple method of mosquito ovary dissection. Florida Entomol. 1968;51(1):31–5.

[74] Burdick DJ, Kardos EH. The age structure of fall, winter, and spring populations of *Culex tarsalis* in Kern Covinty, California. Ann Entomol Soc Am. 1963;56:581–535.

[75] Kardos EH, Bellamy RE. Distinguishing nulliparous from parous female *Culex tarsalis* by examination of the ovarian tracheation. Ann Entomol Soc Am. 1961;54:448–51.

[76] Nelson RL. A comparison of two techniques for distinguishing parous from nulliparous *Culex tarsalis* Coquillett. Mosq New. 1966;26(1):11–3.

[77] Moore CG. Seasonal variation in autogeny in *Culex tarsalis* Coq. in northern California. Mosq News. 1963;23(3):238–41.

[78] Spadoni RD, Nelson RL, Reeves WC. Seasonal occurrence, egg production, and blood-feeding activity of autogenous *Culex tarsalis*. Ann Entomol Soc Am. 1974;67(6):895–902.

[79] Bates D, Mächler M, Bolker B, Walker S. Fitting linear mixed-effects models using lme4. J Stat Softw. 2015;67(1):1–48.

[80] Lesnoff M, Lancelot R. aod: analysis of overdispersed data. 2012. version 1.3.1. Available from: http://cran.r-project.org/package=aod.

[81] Cadmus KJ, Mete A, Harris M, Anderson D, Davison S, Sato Y, et al. Causes of mortality in backyard poultry in eight states in the United States. J Vet Diagnostic Investig. 2019;31(3):318–26. doi: 10.1177/1040638719848718 31084344 PMC6838705

[82] Nolan LK, Vaillancourt J, Barbieri NL, Logue CM. Diseases of Poultry. 14th ed. Swayne DE, Boulianne M, Logue CM, McDougald LR, Nair V, Suarez DL, et al., editors. Diseases of Poultry. Wiley; 2020.

[83] Kobylinski KC, Deus KM, Butters MP, Hongyu T, Gray M, da Silva IM, et al. The effect of oral anthelmintics on the survivorship and re-feeding frequency of anthropophilic mosquito disease vectors. Acta Trop. 2010;116(2):119–26. doi: 10.1016/j.actatropica.2010.06.001 20540931 PMC2939250

[84] Butters MP, Kobylinski KC, Deus KM, da Silva IM, Gray M, Sylla M, et al. Comparative evaluation of systemic drugs for their effects against *Anopheles gambiae*. Acta Trop. 2012 Jan;121(1):34–43. doi: 10.1016/j.actatropica.2011.10.007 22019935 PMC3236608

[85] Cirak V, Aksit D, Cihan H, Gokbulut C. Plasma dispositions and concentrations of ivermectin in eggs following treatment of laying hens. N Z Vet J. 2018 May 4;66(3):121–5. doi: 10.1080/00480169.2018.1426504 29378154

[86] Moreno L, Dominguez P, Farias C, Canton L, Virkel G, Mate L, et al. Ivermectin pharmacokinetics, metabolism, and tissue/egg residue profiles in laying hens. J Agric Food Chem. 2015;63:A–F. 10.1021/acs.jafc.5b04632 26553292

[87] Reisen WK, Milby MM, Reeves WC, Eberle MW, Meyer RP, Schaefer CH, et al. Aerial adulticiding for the suppression of *Culex tarsalis* in Kern County, California, using low volume propoxur: 2. Impact on natural populations in foothill and valley habitats. J Am Mosq Control Assoc. 1985;1(2):154–63. 3880226

[88] Kwan JL, Kluh S, Madon MB, Nguyen D V., Barker CM, Reisen WK. Sentinel chicken seroconversions track tangential transmission of West Nile virus to humans in the greater Los Angeles area of California. Am J Trop Med Hyg. 2010;83(5):1137–45. doi: 10.4269/ajtmh.2010.10-0078 21036853 PMC2963985

[89] Reeves WC, Asman M, Hardy JL, Milby MM, Reisen WK. Epidemiology and Control of Mosquito-borne arboviruses. Sacramento, CA: California Mosquito and Vector Control Association, Inc.; 1990.

[90] Barker CM, Eldridge BF, Reisen WK. Seasonal abundance of *Culex tarsalis* and *Culex pipiens* complex mosquitoes (Diptera: Culicidae) in California. J Med Ent. 2010;47(5):759–68. 10.1603/me09139 PMC2965637 20939368

[91] Nielsen CF, Armijos MV, Wheeler S, Carpenter TE, Boyce WM, Kelley K, et al. Risk factors associated with human infection during the 2006 West Nile virus outbreak in Davis, a residential community in northern California. Am J Trop Med Hyg. 2008 Jan;78(1):53–62. 18187785 PMC2215055

[92] Healy JM, Reisen WK, Kramer VL, Fischer M, Lindsey NP, Nasci RS, et al. Comparison of the efficiency and cost of West Nile virus surveillance methods in California. Vector-Borne Zoonotic Dis. 2015;15(2):147–55. doi: 10.1089/vbz.2014.1689 25700046 PMC4340646

[93] Chakraborty S, Smith RL. Error associated with estimates of minimum infection rate for endemic West Nile Virus in areas of low mosquito trap density. Sci Rep. 2019;9(1):1–8. doi: 10.1038/s41598-018-37186-2 31836789 PMC6911069

[94] Trott KA, Giannitti F, Rimoldi G, Hill A, Woods L, Barr B, et al. Fatty liver hemorrhagic syndrome in the backyard chicken: A retrospective histopathologic case series. Vet Pathol. 2014;51(4):787–95. doi: 10.1177/0300985813503569 24091813

[95] California Department of Public Health Vector-Borne Disease Section. California Arbovirus Surveillance Bulletins. Richmond, CA; Available from: https://westnile.ca.gov/resources_reports.php?report_category_id=6#nav-1-1-default-hor-left-rounded-underline—9.

[96] Polovodova VP. The determination of the physiological age of female *Anopheles* by the number of gonotrophic cycles completed. Medskaya Parazit. 1949;18:352–5.

[97] Nelson RL. Parity in winter populations of *Culex tarsalis* coquillett in Kern county, California. Am J Epidemiol. 1964;80(2):242–53. doi: 10.1093/oxfordjournals.aje.a120473 14215835

[98] Pezzin A, Sy V, Puggioli A, Veronesi R, Carrieri M, Maccagnani B, et al. Comparative study on the effectiveness of different mosquito traps in arbovirus surveillance with a focus on WNV detection. Acta Trop. 2016;153:93–100. doi: 10.1016/j.actatropica.2015.10.002 26466982

[99] R P. Malaria in the Jeypore Hill Tract and adjoining coastland. Paludism. 1912;5:32.

[100] Gray L, McCabe R, Asay B, Kroma B, Sougue ED, Some AF, et al. No need to wing it: a new method for quickly and accurately age-grading mmosquitoes utilizing wing morphology. virtual: American Society of Tropical Medicine and Hygiene; 2020.

[101] Canga AG, Prieto AMS, Liebana MJD, Martinez NF, Vega MS, Vieitez JJG. The pharmacokinetics and metabolism of ivermectin in domestic animal species. Vet J. 2009;179(1):25–37. doi: 10.1016/j.tvjl.2007.07.011 17851096

[102] Keukens HJ, Kan CA, van Rhijin JA, van Dijk J. Ivermectin residues in eggs of laying hens and in muscle and liver of broilers after administration of feeds containing low levels of ivermectin. Proc Euroresidue IV Conf Residues Vet Drugs Food. 2000;(May 2000):678–82.

[103] Kaplan RM, Vidyashankar AN. An inconvenient truth: Global worming and anthelmintic resistance. Vet Parasitol. 2012;186(1–2):70–8. doi: 10.1016/j.vetpar.2011.11.048 22154968

[104] Geerts S, Gryseels B. Drug resistance in human helminths: Current situation and lessons from livestock. Clin Microbiol Rev. 2000;13(2):207–22. doi: 10.1128/CMR.13.2.207 10755998 PMC100151

[105] Wolstenholme AJ, Evans CC, Jimenez PD, Moorhead AR. The emergence of macrocyclic lactone resistance in the canine heartworm, *Dirofilaria immitis*. Parasitology. 2015;142(10):1249–59. doi: 10.1017/S003118201500061X 26040450

[106] Abbas RZ, Zaman MA, Colwell DD, Gilleard J, Iqbal Z. Acaricide resistance in cattle ticks and approaches to its management: The state of play. Vet Parasitol. 2014;203(1–2):6–20. doi: 10.1016/j.vetpar.2014.03.006 24709006

[107] Nicolas P, Kiuru C, Wagah MG, Muturi M, Duthaler U, Hammann F, et al. Potential metabolic resistance mechanisms to ivermectin in *Anopheles gambiae*: a synergist bioassay study. Parasites and Vectors. 2021;14(1):1–12. doi: 10.1186/s13071-020-04505-4 33743783 PMC7981804

[108] Coccini T, Candura SM, Manzo L, Costa LG, Tonini M. Interaction of the neurotoxic pesticides ivermectin and lindane with the enteric GABAA receptor-ionophore complex in the guinea-pig. Eur J Pharmacol Environ Toxicol. 1993;248(1):1–6. doi: 10.1016/0926-6917(93)90018-l 7687958

[109] Tonini M, Costa LG, Candura SM, Olibet G, Rizzi CA, Garlaschelli L, et al. Interaction of the pyrethroid insecticides tetramethrin and cypermethrin with enteric cholinergic transmission in the guinea-pig. Neurotoxicology. 1989;10(4):707–15. 2562766

