# Supplementary figures and images for: Effects of ivermectin treatment of backyard chickens on mosquito dynamics and West Nile virus transmission
Source: PLoS Negl Trop Dis. 2022 Mar 25;16(3):e0010260. doi: 10.1371/journal.pntd.0010260 (PMC9012369; doi:10.1371/journal.pntd.0010260)

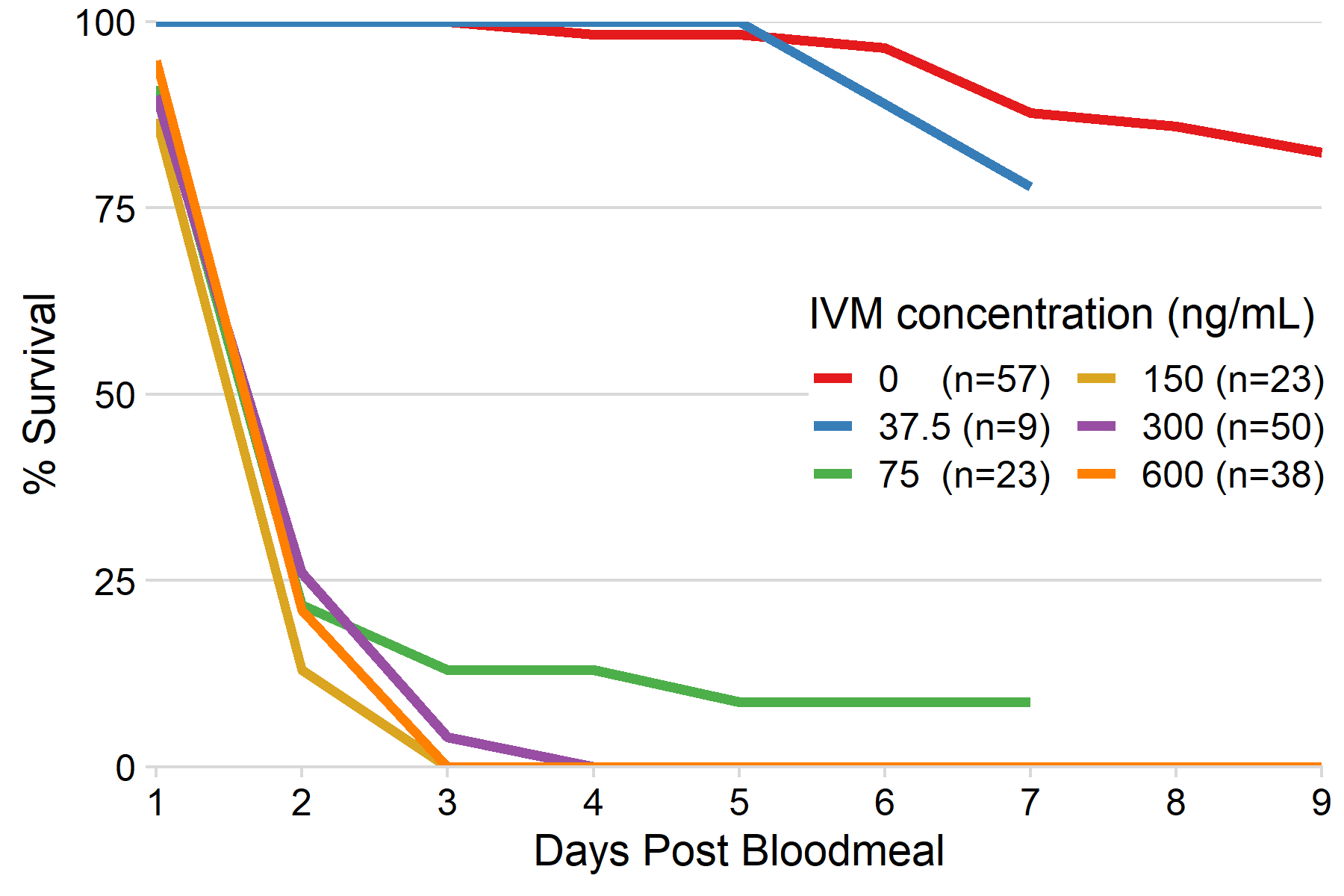

Supplement: S1 Fig — Cx. tarsalis (Kern Natural Wildlife Reserve colony) survival following a membrane bloodmeal containing serial dilutions of IVM. Number of blood-fed female mosquitoes at each IVM concentration indicated. (TIF) [file pntd.0010260.s001.tif]

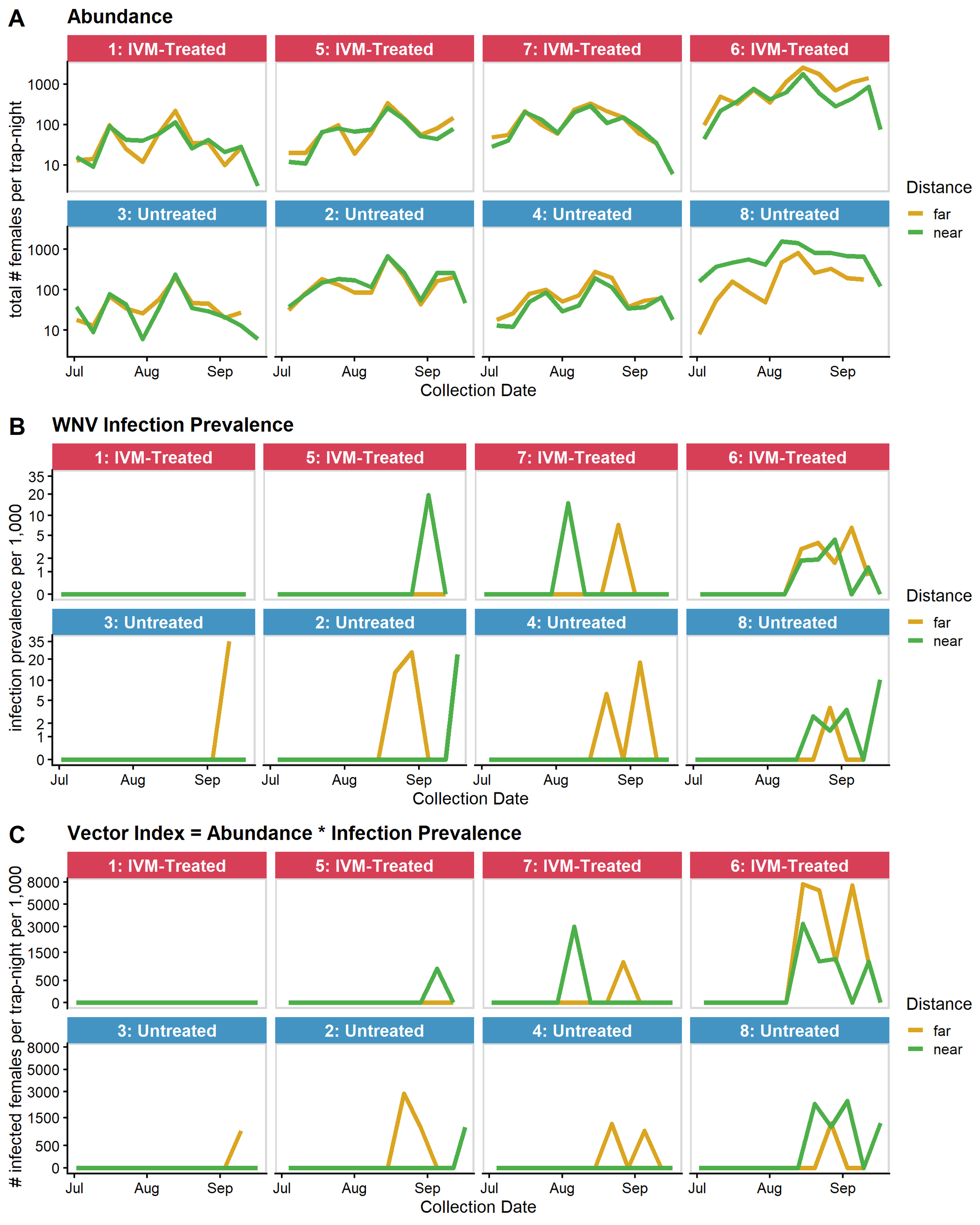

Supplement: S2 Fig — Weekly (A) abundance, (B) infection prevalence per 1,000, and (C) vector index (VI) near (≤ 10m) and far (~150m) from ivermectin (IVM)-treated and untreated flocks. VI is a risk metric that approximates the number of infectious mosquitoes present as the product of abundance and infection rate. Individual plot headers indicate site number (see Fig 1) and treatment status and are ordered by spatial location west to east (L to R). (TIF) [file pntd.0010260.s002.tif]
