# Supplementary material for: Effects of ivermectin treatment of backyard chickens on mosquito dynamics and West Nile virus transmission
Source: PLoS Negl Trop Dis. 2022 Mar 25;16(3):e0010260. doi: 10.1371/journal.pntd.0010260 (PMC9012369; doi:10.1371/journal.pntd.0010260)
Supplement: S1 Table — Fixed and random effect estimates from mixed effects logistic regression for parity in Cx. tarsalis mosquitoes at near and far distances from ivermectin (IVM)-treated and untreated control flocks. (DOCX) [file pntd.0010260.s003.docx]

**S1 Table**. **Final model estimates.**

|  |  | **Estimate** | **Standard Deviation** | ***P-*value** |
| --- | --- | --- | --- | --- |
| **Fixed Effects** | **Intercept** | -0.475 | 0.162 | 0.003 |
|  | **Control-far** | 0.085 | 0.103 | 0.407 |
|  | **IVM-far** | -0.127 | 0.095 | 0.184 |
|  | **IVM-near** | -0.301 | 0.099 | 0.002 |
| **Random Effects*** | **Week 29** | 0.036 | 0.118 | NA |
|  | **Week 30** | -0.456 | 0.150 |  |
|  | **Week 31** | -0.348 | 0.139 |  |
|  | **Week 32** | -0.419 | 0.108 |  |
|  | **Week 33** | -0.414 | 0.073 |  |
|  | **Week 34** | 0.267 | 0.085 |  |
|  | **Week 35** | -0.0004 | 0.122 |  |
|  | **Week 36** | 0.670 | 0.104 |  |
|  | **Week 37** | 0.679 | 0.102 |  |

Fixed and random effect estimates from mixed effects logistic regression for parity in *Cx. tarsalis* mosquitoes at near and far distances from ivermectin (IVM)-treated and untreated control flocks.

* Random intercept by week of the year
