# Supplementary material for: Effects of ivermectin treatment of backyard chickens on mosquito dynamics and West Nile virus transmission
Source: PLoS Negl Trop Dis. 2022 Mar 25;16(3):e0010260. doi: 10.1371/journal.pntd.0010260 (PMC9012369; doi:10.1371/journal.pntd.0010260)
Supplement: S2 File — Applications occurred June 15-Sept 25, 2019 by Sacramento-Yolo Mosquito and Vector Control District within 2 km of the city limits of Davis, California. Table A: Total ground-based spray events within 500m of each coop (June 15-Sept 25). Table B: Ground-based sprays within 500m of coops, occurring early and late in the study period. (DOCX) [file pntd.0010260.s013.docx]

**S2 File. Organized ground and aerial-based insecticide applications during study period.** Applications occurred June 15-Sept 25, 2019 by Sacramento-Yolo Mosquito and Vector Control District within 2 km of the city limits of Davis, California.

The local vector control district (Sacramento-Yolo Mosquito and Vector Control District, SYMVCD) performed both aerial (*n* = 40) and ground-based (*n* = 40) applications of insecticides to control adult mosquitoes in and near (≤ 2 km) the City of Davis during the study period. Standard operating procedures for SYMVCD is to follow up on positive mosquito pools with vector control and proactively manage populations to prevent viral amplification.

From June 15-Sept 25, there were an average of 3.5 applications within 500 m of treated flocks and 5.25 within 500 m of untreated flocks (Table A). There was no difference in number of applications around coops by treatment status (*P* = 0.741). The closest aerially sprayed area was ≥1,000 m from all coops.

**Table A. Total ground-based spray events within 500m of each coop (June 15-Sept 25).**

| **Coop** | **IVM-Treated** | | **Untreated** | ***P-*value*** |
| --- | --- | --- | --- | --- |
| Coop 1 | 0 | - | | 0.741 |
| Coop 2 | - | 21 | |  |
| Coop 3 | - | 0 | |  |
| Coop 4 | - | 0 | |  |
| Coop 5 | 0 | - | |  |
| Coop 6 | 13 | - | |  |
| Coop 7 | 1 | - | |  |
| Coop 8 | - | 0 | |  |
| **Total** | **14** | **21** | |  |

*Asymptotic Wilcoxon-Mann-Whitney *P*-value.

There were more applications during the latter half of the study (after the Aug 13 midpoint of the study), but there was no difference in distribution of sprays across the earlier and later timeframes between treatment status (*P* = 0.551; Table B).

**Table B. Ground-based sprays within 500m of coops, occurring early and late in the study period.**

|  | **Time of Spray** | | | **­*P*-value*** |
| --- | --- | --- | --- | --- |
| **Treatment Status** | **Early^#^** | **Late^+^** | **Total** |  |
| IVM-Treated | 2 | 12 | 14 | 0.551 |
| Untreated | 1 | 20 | 21 |  |

^#^Sprays occurring during the first half of the study (up to Aug 13, 2019).

^+^Sprays occurring during the second half of the study (after Aug 13, 2019).

*Fisher exact *P*-value.
